# Towards computerizing intensive care sedation guidelines: design of a rule-based architecture for automated execution of clinical guidelines

**DOI:** 10.1186/1472-6947-10-3

**Published:** 2010-01-18

**Authors:** Femke Ongenae, Femke De Backere, Kristof Steurbaut, Kirsten Colpaert, Wannes Kerckhove, Johan Decruyenaere, Filip De Turck

**Affiliations:** 1 Department of Information Technology (INTEC), Ghent University - IBBT, Gaston Crommenlaan 8, Bus 201, 9050 Ghent, Belgium; 2 Department of Intensive Care, Ghent University Hospital, De Pintelaan 185, 9000 Ghent, Belgium

## Abstract

**Background:**

Computerized ICUs rely on software services to convey the medical condition of their patients as well as assisting the staff in taking treatment decisions. Such services are useful for following clinical guidelines quickly and accurately. However, the development of services is often time-consuming and error-prone. Consequently, many care-related activities are still conducted based on manually constructed guidelines. These are often ambiguous, which leads to unnecessary variations in treatments and costs.

The goal of this paper is to present a semi-automatic verification and translation framework capable of turning manually constructed diagrams into ready-to-use programs. This framework combines the strengths of the manual and service-oriented approaches while decreasing their disadvantages. The aim is to close the gap in communication between the IT and the medical domain. This leads to a less time-consuming and error-prone development phase and a shorter clinical evaluation phase.

**Methods:**

A framework is proposed that semi-automatically translates a clinical guideline, expressed as an XML-based flow chart, into a Drools Rule Flow by employing semantic technologies such as ontologies and SWRL. An overview of the architecture is given and all the technology choices are thoroughly motivated. Finally, it is shown how this framework can be integrated into a service-oriented architecture (SOA).

**Results:**

The applicability of the Drools Rule language to express clinical guidelines is evaluated by translating an example guideline, namely the sedation protocol used for the anaesthetization of patients, to a Drools Rule Flow and executing and deploying this Rule-based application as a part of a SOA. The results show that the performance of Drools is comparable to other technologies such as Web Services and increases with the number of decision nodes present in the Rule Flow. Most delays are introduced by loading the Rule Flows.

**Conclusions:**

The framework is an effective solution for computerizing clinical guidelines as it allows for quick development, evaluation and human-readable visualization of the Rules and has a good performance. By monitoring the parameters of the patient to automatically detect exceptional situations and problems and by notifying the medical staff of tasks that need to be performed, the computerized sedation guideline improves the execution of the guideline.

## Background

### Introduction

Computerized decision support systems (CDSS) have the potential of improving the quality of health care [1-3]. However, more than 20 years after the first reports about this potential, the adoption rate of using Information and Communication Technology (ICT) to quickly and accurately guide diagnosis and therapy is still very low. However, one of the main reasons for this slow adoption rate is the gap in communication between the ICT and medical domain. These projects unite people with different backgrounds, such as software developers, health services researchers, physicians and domain experts. Uniting all these people in a team requires effort and commitment to overcome the communication problems caused by the gap between the information and knowledge necessary to implement guidelines and the information used in guidelines. The developers often do not have the required medical domain knowledge, thus a lot of evaluation and testing is needed to make sure the service works correctly. The evaluation is also hampered by the fact that the medical staff does not understand the code of the program as it is not presented in a human-readable format. Moreover, clinicians sometimes doubt the effectiveness of the use of a CDSS [3]. This leads to a very time-consuming and error-prone development and often results in inconsistencies [4]. This problem can be approached by using bridge personnel who have the knowledge of multiple disciplines used in this process [5]. However, this personnel is often difficult to find. As a consequence, a lot of care-related activities are still being conducted based on manually constructed guidelines or flow charts. Such a flow chart contains medical instructions for diagnosis, treatment and follow-up of a certain medical condition. However, the key problem encountered here is that such schemes are often ambiguous, simplified, incomplete and fail to cover all situations that may occur [6-8]. This leads to unnecessary variations in treatments. The flow charts lack definitions, focus on omission errors, timing of events, and concurrent drug therapy [9]. The conditions used in the guideline fail to specify the parameters on which the decisions are based. These variables are often described at the wrong level of abstraction. Moreover, the actions may not be suitable for execution and are sometimes too abstract [5]. The sedation guideline, used for the depression of consciousness of critically ill patients, is an example of a complex guideline which is implemented in the Intensive Care Units (ICUs) by providing the medical staff with manually constructed flow charts. These flow charts contain many ambiguities and simplifications and do not enforce correct and timely execution of the guideline.

Kawamoto et al [10] argues that automatically providing decision support as part of the clinicians workflow is the most important and effective feature of CDSS to modify the behavior of clinicians. Other factors to improve the use of decision support systems are: providing support at the time and location of decision making, giving a recommendation and using a computer [11,12]. Thus, a semi-automatic verification and translation framework, capable of turning manually constructed flow charts into ready to use programs, would combine the strengths of service-oriented and manual approaches while decreasing their disadvantages.

**Figure 1:**
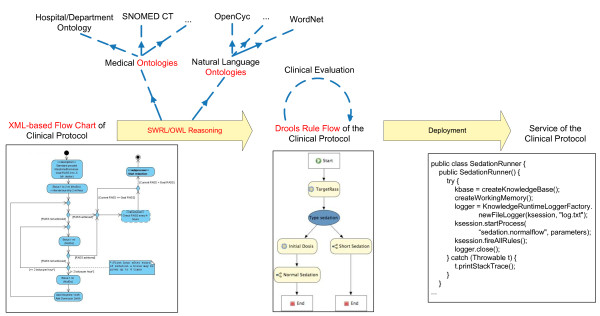
**High level overview of the framework to semi-automatically translate clinical guidelines**.

The ICU of the Ghent University Hospital is currently evaluating a service-oriented platform, the Intensive Care Service Platform (ICSP) [13], that supports physicians in the follow-up of patients by providing a number of medical support services that monitor the condition of the patient and make medical suggestions or produce new data that can be used by other services. In this paper we propose an extension of this platform with an architecture that semi-automatically translates the XML-based [14] flow charts into Drools Rule Flows [15] by employing semantic technologies such as ontologies [16] and the Semantic Web Rule Language (SWRL) [17]. The complete flow of the proposed solution is depicted in Figure 1. The translation process will focus on guidelines represented as XML-based flow charts. The ontologies encode the medical and natural language domain knowledge which can occur in the flow charts. SWRL is used to write Rules on top of these ontologies. Reasoning on top of these ontologies and the Rules are responsible for the translation of the information in the XML flow charts to the Drools Rule language. Eventually a computerized clinical guideline is obtained which is deployed on the ICSP platform as a service and gives notifications to the medical staff about tasks that need to be performed or problems that are detected. The goal of this paper is two-fold. On the one hand, a conceptual description of a semi-automatic translation framework capable of turning a clinical guideline, expressed as a flow chart, into a working Rule-based application is presented. On the other hand, the applicability of the Drools Rule language to computerize clinical guidelines is evaluated by expressing an example guideline, namely the sedation protocol, as a Drools Rule Flow and executing and deploying this Rule-based application as a part of the ICSP platform.

### Related Work

In this section we present some of the research literature related to computerizing clinical guidelines, Rule-based systems, ontologies and the ICSP platform.

#### Computerizing clinical guidelines

A lot of standardization efforts and formalisms for representing clinical guidelines have been proposed in literature [18-21]. The most prevalent formats are the Arden Syntax [22,23], PROforma [24,25], EON [26], GLIF [27,28], PRODIGY [29], Asbru [30] and Guide [31]. More information about these formats can be found in Additional file 1.

Some research has also been done on automatically translating manually constructed guidelines to computer programs. Kaiser et al [32] propose a multi-step approach using information extraction and transformation to extract process information from clinical guidelines. Heuristics were applied to perform this extraction. Using patterns in the structure of the document and in expressions, the need of natural language processing (NLP) was eliminated. This approach differs from our approach as it only works on very structured documents. No interaction with a domain expert occurs, which heightens the chance of errors in the translation.

#### Rule-based systems

Rule-based systems [33] are used in the field of Artificial Intelligence (AI) to represent and manipulate knowledge in a declarative manner. The domain-specific knowledge is described by a set of (production) rules in the production memory. A Rule can be seen as a simple mathematical implication of the form A → C, where A is the set of conditions, or antecedent, and C is the set of actions to be taken, or consequent. The general idea is that a Rule-based system holds a predefined set of Rules in its memory. From that moment on, a large number of Facts, which represent the data in the *Working memory*, can be given to the engine. It will check the conditions of each Rule against the Facts. If all the conditions of a Rule are said to be valid, it is fired. When a Rule is fired, its predefined consequent will be executed. 

*Rule Engines *require extensive pattern matching during their execution. It has been estimated that up to 90% of a *Rule Engine's *run time is spent on performing repetitive pattern matching between the Rule set and the working memory elements [34]. Originally String comparison algorithms were used for this such as Boyer-Moore, Knuth-Morris-Pratt and Rabin-Karp. In 1974 the Rete algorithm was published by Dr. Charles L. Forgy [35]. It makes the Rule-to-Fact matching process a lot quicker than the previously mentioned algorithms. When Rules are added, the Rete algorithm constructs a network of nodes, each representing a pattern from the conditions of the Rules. These nodes are connected with each other, whenever the corresponding patterns are in the same antecedent of one of the Rules. The constructed network looks like a tree, with the leaves being the consequents of the Rules. If a path is traced from the root node all the way to one of the leaves, a complete Rule is described. When the Facts are added to the algorithm, they are placed in memory next to each node where the pattern matches the Fact. Once a full path, from root to leaf, is described, a Rule is fired and its consequent is executed.

#### Ontologies

Ontologies [16] can structure and represent knowledge about a certain domain in a formal way. This knowledge can then easily be shared and reused. The Ontology Web Language (OWL) is the leading language for encoding these ontologies. Because of the foundation of OWL in first-order logic, the models and description of data in these models can be formally proved. It can also be used to detect inconsistencies in the model as well as infer new information out of the correlation of this data. This proofing and classification process is referred to as Reasoning. Reasoners are implemented as generic software-modules, independent of the domain-specific problem. For this research, the Reasoner Pellet [36] was used. Existing medical and natural language ontologies can be used to support the translation process. Cyc [37,38] and WordNet [39] are two well-known ontologies that model general knowledge about the English language such as synonyms and generally true statements. More information about these ontologies can be found in Additional file 2. A wide range of ontologies exist about the eHealth domain. Additional file 2 gives an overview of the most relevant, well-known and well-developed eHealth ontologies, which are available in OWL and that could be (partially) reused to support the semi-automatic translation such as LinkBase [40,41], SNOMED CT [42-44], the Galen Common Reference Model [45,46], the NCI Cancer Ontology [47,48], the Foundational Model of Anatomy Ontology (FMA) [49,50], the Gene Ontology (GO) [51,52] and the Ontology for Biomedical Investigations (OBI) [53,54].

#### Intensive Care Service Platform (ICSP)

The successful use of CDSS requires structured and standardised information in the Electronic Health Record (EHR). However, EHR has some limitations, such as clinical data limitations (different meaning of words), technological limitations (usage on PDAs, interoperability) and the lack of standardization [55,56]. Another problem is that less than 20% of the hospitals are completely digital while access through an electronic platform is necessary to adopt CDSS [57,58].

The computerization of the Intensive Care Unit of Ghent University hospital was started in 2003 by implementing a system, the ICSP platform, which gathers all generated patient data and stores it in a large database called IZIS (Intensive Care Information System). The ICSP platform consists of a number of services. A bed-side PC allows the medical staff to input clinical observations and prescription and administration of medication. Monitor parameters, administrative data and results of medical tests are automatically gathered from monitoring equipment and other databases. Services monitor the condition of the patient and suggest medical decisions or produce new data which is stored in the database and can be used by other services.

The ICSP platform is an example of a Service-Oriented Architecture (SOA) [59]. The main idea behind SOA is the separation of the functions of the system into well-defined, independent, reusable and distributable components, referred to as services. These services communicate with each other by passing data from one service to another or by coordinating an activity between two or more services. Web Services [60] are often used to implement this architecture.

### Paper Organization

The remainder of this article is organized as follows. The Methods section begins with an overview of the high level architecture of the platform. Next, the choice of Drools as the ideal Rule language for implementing clincical guidelines is motivated. Thereafter, an architecture for semi-automatically translating the XML-based flow charts to Drools Rule Flows is detailed. Next, a description of the platform that integrates this Rule-based system into a Service-Oriented Architecture (SOA) is given. This section ends with an overview of the methods that were used to evaluate the proposed platform, namely the BMI application and the ICU sedation guideline. The Results section further investigates the suitability of the Drools Rule language for the implementation of clinical guidelines by exploring its performance. The results of translating the ICU use case, the sedation guideline, are also detailed. Finally the main conclusions of this research are discussed and highlighted.

## Methods

### High-level architecture

**Figure 2:**
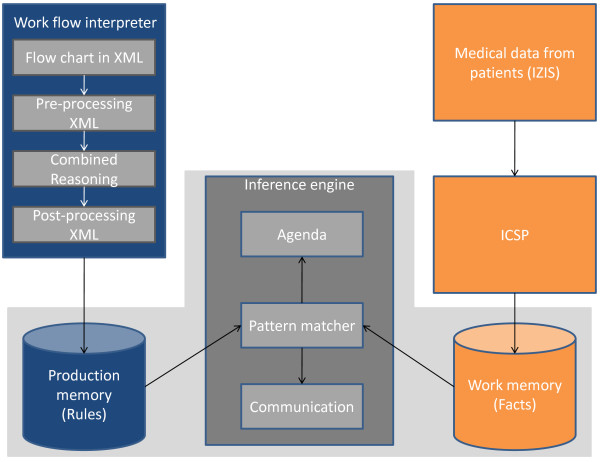
**High level overview of the architecture**. Gives a high level overview of the complete architecture. The top left part of the figure represents the components responsible for translating XML-based flow charts into Drools Rule Flows. These components were designed by the authors. The components surrounded by a gray shade visualize the different modules of a *Rule-based system *such as Drools. These are external components which exist independently. Every possible *Rule Engine *could be inserted as long as a *Post-processing *module exists for it. This module translates the intermediate language to the specific language of the Rule Engine. The authors have designed a Post-processing module for Drools. The components at the top right of the figure illustrate the communication of the proposed architecture with the existing *ICSP platform*. The *ICSP platform *and the IZIS database were constructed by the co-authors.

A high level overview of the architecture of the platform is shown in Figure 2. The components at the top left of Figure 2 are responsible for the semi-automatic translation of the clinical guideline into a Drools Rule Flow. The XML-based flow chart, designed by the medical staff, enters the platform in the *Workflow Interpreter Component*. All the excess XML-code is removed by the *Pre-processing module *to ideally prepare the flow chart for the translation. Then the *Combined Reasoning (CR) module *processes and translates the flow chart to an intermediate language by employing ontologies and SWRL Rules. The *Post-processing module *delivers a working application by translating this intermediate language to a Drools Rule Flow. These components are discussed in more detail in the section on semi-automatic translation. 

The components at the top right of Figure 2 illustrate the input of medical data about patients. This data is collected by querying the IZIS database through the *ICSP platform *and communicating with the nursing staff. More information about these components, which were constructed by the co-authors, can be found in the Related Work section and the section on integrating a Rule-based system into a service-oriented platform.

The components surrounded by a gray shade represent a Rule-based system which is responsible for the correct execution of the application. These components exist independently and are further explored in the following section. The flow (the Rules) is loaded into the *Production memory *and the Facts (the medical data) in the *Working memory*. Next, the *Inference engine *will execute the clinical guideline by employing a *Pattern matcher*. This module matches the Rules on Facts. When a match is found, the parameters in the Rule are substituted by the Facts and the Rule is executed. As a consequence, new Rules can be fired. Inputting additional Facts can also trigger the execution of Rules. This way the entire clinical guideline is executed. The *Agenda module *is responsible for ordering the Rules. When more than one Rule can be fired at the same time, the one with the highest priority will be fired first.

Sometimes the execution of a Rule requires communication with the medical staff, for example when additional medical input is needed or when the staff needs to be made aware that a task has to be executed e.g. giving medication. This is handled by the *Communication module*.

Note, that this component-based architecture is very flexible and adaptable. One can, for example, easily opt to use another Rule language by writing a new *Post-processing *module and plugging it into the platform.

### Rule-based execution environment: technology choices

In this section, we concentrate in particular on the bottom part of Figure 2 which is surrounded by a gray shade. A *Rule Engine *was chosen to implement and deploy the clinical guideline instead of using one of the mentioned representation formats developed specifically for computerizing clinical guidelines (see Related Work section). The greatest benefit offered by the Rule-based approach is that by separating the domain-specific knowledge (described by the Rules) from the implementing logic, it becomes possible to alter and extend the provided functionality at runtime. Some of the formats, such as GLIF and Guide, have very sophisticated and complex representations which makes it very difficult to semi-automatically translate to these formats. These formats also focus on a specific application domain, namely medical guidelines. Rules on the other hand have a simple representation format, which allows to easily express and evaluate difficult problems. They are also more easily interpretable by domain experts than hundreds of lines of programming language code. Moreover, *Rule Engines *can be used to represent guidelines for a wide variety of purposes. This way the semi-automatic translation framework can be easily employed in other application domains, such as helpdesk guidelines.

However, the current framework could easily be adapted to use one of the mentioned representation formats for computerizing clinical guidelines. A new *Post-processing *module needs to be written that translates the intermediate language, which is outputted by the *CR *module, to the representation language of the chosen clinical guideline format.

The remainder of this subsection is organized as follows. First, the desired features, which the chosen *Rule Engine *should have, are determined. Based on these, the choice for Drools as the ideal *Rule Engine *to express and semi-automatically translate clinical guidelines is motivated.

#### Comparison of existing, prevalent Rule Engines

For comparing the different studied *Rule Engines*, the following arguments, in order of importance, were taken into account:

• Nowadays most *Rule Engines *employ the Rete algorithm (or a variant of it) (see Related Work section). Since the publication of this algorithm not a lot of improvements have been made. Optimizations of Rete (such as Leaps, Treats and Rete II) have been proposed which perform better in very specific situations. However, Rete is still the leading algorithm for general-purpose *Rule Engines*. Moreover, the Rete algorithm sacrifices memory for speed. Since speed is of critical importance in medical applications, *Rule Engines*, which implement this algorithm, are preferred.

• An important goal of this work is to close the gap in communication between the domain experts, e.g. the medical staff, and the IT developers. *Rule Engines *are favored which are backed with tools that support this cause, such as user-friendly Rule Editor Graphical User Interfaces (GUIs) and explanation features that visualize how a certain conclusion was obtained by the Rules.

• A guideline flow chart is in essence a workflow as it expresses a sequence of steps, such as actions and decisions that need to be taken. Therefore, *Rule Engines *are favored for which an existing integration with a workflow engine exists. This allows to more easily translate the guideline to this format which closely resembles the original flow chart.

• To ease the understanding of the *Rule Engine *and its integration into the *ICSP platform*, it is desired to be Open Source.

• Platform and database independent *Rule Engines *are preferred.

• Actively maintained *Rule Engines*, with a large user base and good documentation are favored.

• *Rule Engines *which offer additional tools, e.g. for testing or persisting the Rules and Facts, are desired.

A list of possible *Rule Engines*, including for example Mandarax [61], Jess [62], Biztalk Server [63] and ILog JRules [64], was investigated. Eventually, JBoss Drools [15], a free, Open Source, Java-based *Rule Engine*, was chosen. It provides an implementation of the Rete algorithm [65], the ReteOO [66] algorithm, which is basically an improved version of Rete that takes advantage of the fact that nowadays programming languages are object-oriented. Moreover, Drools offers a component, the Drools Flow engine [67], that allows to implement Rules in the shape of flow charts. This closely resembles the format of the original clinical guideline XML-based flow chart. This helps to close the gap in communication as the Drools flow charts can easily be visualized to the medical staff for evaluation. Drools Rule Flow files are internally stored in XML, which additionally eases the automatic translation. On top of this, there exists a Drools plug-in for the Eclipse platform [68] that offers a wide range of tools for debugging, testing, editing, visualizing and persisting the Rules. Drools is actively maintained, has a very large user community and provides solid documentation.

#### Drools

**Figure 3:**
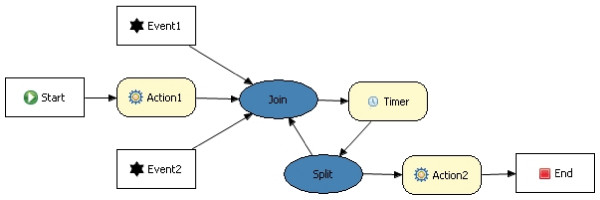
**A simple example of a Drools Rule Flow**.

The capabilities of Drools were studied in depth to make sure it was a suitable Rule language to express clinical guidelines. As we mentioned in the previous section, the presence of the Rule Flow component was an important argument to choose Drools. This is a work flow or process engine which allows advanced integration of Rules and processes. A Rule Flow describes the order in which a series of steps should be executed, by using a flow chart with an XML-based encoding. A simple example can be seen in Figure 3. Drools provides nodes which implement the *Split *of a path (decision nodes), the *Join *of 2 separate paths and *Loops*. It also provides a *Timer *node, which allows triggering an event after a certain amount of time has passed. This is a very important feature for clinical guidelines as they often contain actions that need to be performed after a certain amount of time or on a regular basis.

It is important that we can interact with the human user as a consequence of a Rule. The *HumanTask *node of the Drools Rule Flow represents an atomic task that has to be executed by a human interacting with the application. It can thus be used to communicate with the medical staff. When a *HumanTask *node is encountered, the flow will only continue when this task is completed. Although this node has all the features we require, it is a fairly new feature in Drools Rule Flow. It was not completely implemented yet. This problem was mediated by using another type of node, namely *Work Items*. This is a very abstract node which can be used by the developers to implement any type of needed action which is not yet included in Drools Flow 5 Milestone 5.

### Semi-automatic translation

This section further details the semi-automatic translation of the XML-based flow chart of the clinical guideline into a Drools Rule Flow. This translation is handled by the *Workflow Interpreter*, the blue box at the top left of Figure 2.

A conceptual architecture for this *Workflow Interpreter *was created using the Attribute Driven Design (ADD) method [69]. ADD works in terms of iterations by utilizing a divide-and-conquer strategy. First, a single module that comprises the entire system is created. Next, it is decomposed into smaller subsystems. The most important quality attribute for this application is modifiability, with usability following shortly behind.

The first ADD iteration, which is represented by the blue box at the top left of Figure 2, is characterized by a pipes and filters pattern. This pattern bears close resemblance to a pipeline where the data flows from one end to another. Filters can be added, replaced or moved by placing pipes accordingly. The use of this pattern guarantees modifiability as new components (filters) can easily be plugged into the system. The XML-based flow chart, designed by the medical staff, enters the platform in the *Workflow Interpreter Component*. All the excess XML-code will be removed by the first filter, namely the *Pre-processing module*, to ideally prepare the flow chart for the translation. Then the *Combined Reasoning (CR) filter *will process and translate the flow chart to an intermediate language. The last filter, the *Post-processing module*, delivers a working application by translating this intermediate language to a Drools Rule Flow. To achieve this, algorithms will be implemented using the advantages and properties of this intermediate XML format. This way, the different components can be detected and the essential code, necessary for the Drools execution engine, can be generated. General handler components will be used which implement a general functionality, such as an interaction component to communicate with the nursing staff or a data component to execute a database query. For example, in the interaction component, only the message needs to be replaced, the rest of the code stays the same for each interaction component.

**Figure 4:**
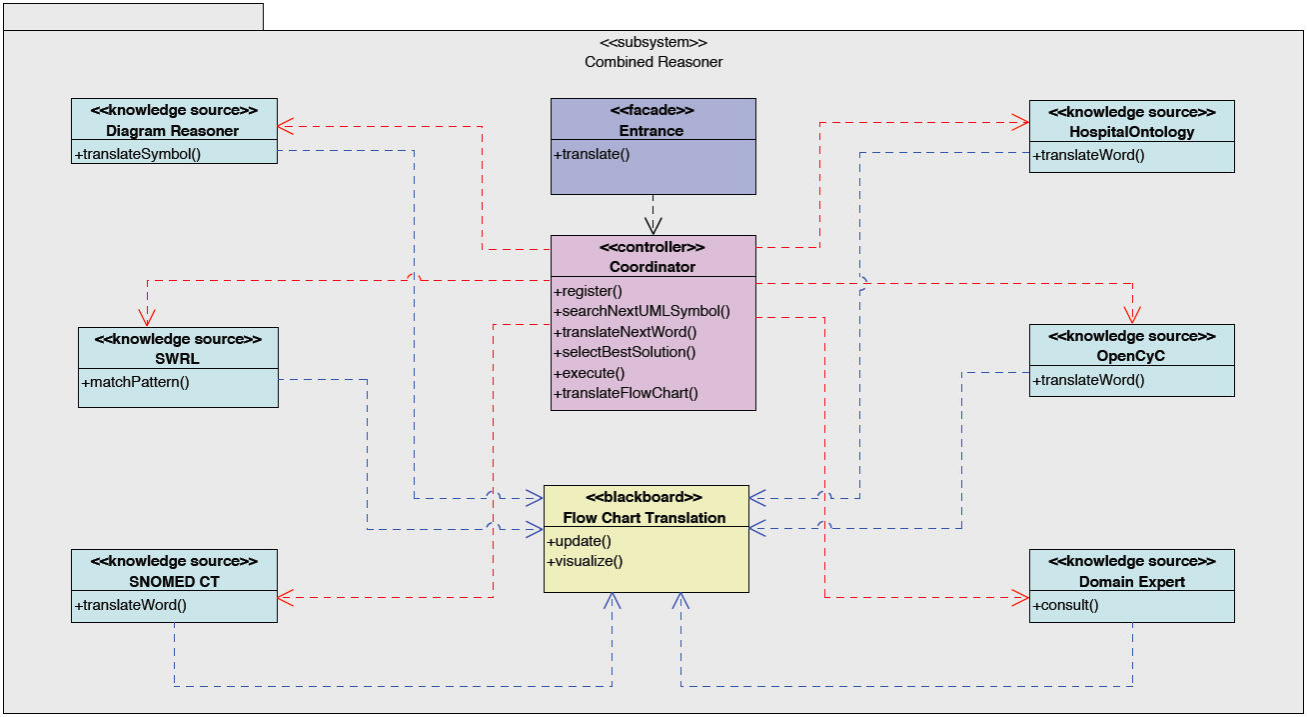
**The class diagram of the Combined Reasoning (CR) module**. Visualizes the second ADD iteration of the semi-automatical translation architecture. It represent the *Combined Reasoning module *through the blackboard pattern. The light blue classes represent *Knowledge Sources*, such as the ontologies with accompanying SWRL Rules and the *Diagram Reasoner*. The yellow class represents the *Blackboard*, which contains the flow chart that is currently being translated. The purple class represent the *Coordinator *which organizes all the activity. The dark blue class functions as the general entry point to the *Combined Reasoning module *and contains methods that can be called by other modules.

The *Combined Reasoning module*, where the bulk of the translation takes place, is molded further in the second ADD iteration through the blackboard pattern, as can be seen in Figure 4. As the name suggests, one can think of a chalkboard in a class room (the *Blackboard *[70]), where students *(Knowledge Sources) *contribute their expertise on the contents of the chalkboard. The teacher *(the Coordinator) *guarantees that things happen in an orderly fashion by allowing only one student at the same time to write on the chalkboard. Moreover, students always communicate indirectly via the chalkboard, never through direct conversation.

This pattern is ideal to apply here because there is not one algorithm that can completely handle the translation process. There are however different heuristics and algorithms available that offer a partial solution to the problem which can be employed as *Knowledge Sources*. New algorithms can easily be plugged into the system which again improves the modifiability of the system.

As *Knowledge Sources*, existing medical and natural language ontologies can be used [71]. Some interesting ontologies are described in the Related Work section. Additionally an ontology can be constructed that models information-specific for the hospital or department where the application will be deployed. The semantics of a flow chart are not only contained within the text, but also in the usage of different symbols and figures. For example, a trapezoid can depict an if-structure. The *Diagram Reasoner Knowledge Source *is used to translate these symbols to the intermediate language. It also employs an ontology, namely the Diagram Ontology, which models information about the used symbols, both in the UML and the intermediate format.

Rules are specified on these ontologies by using SWRL [17]. OWL Reasoning and these SWRL Rules model how the translation can take place. Examples of such Rules can be found in the Results section in the ICU use case: sedation guideline subsection.

A human interactor will have to be involved in this translation process as *Knowledge Source *to solve problems that may occur such as picking between two possible situations, clarifying a symbol by giving a synonym and so on. This human interactor can easily be a medical domain expert such as a physician or a nurse as these questions will be posed in a natural language. This helps to close the gap in communication between the medical staff and the computer scientist.

**Figure 5:**
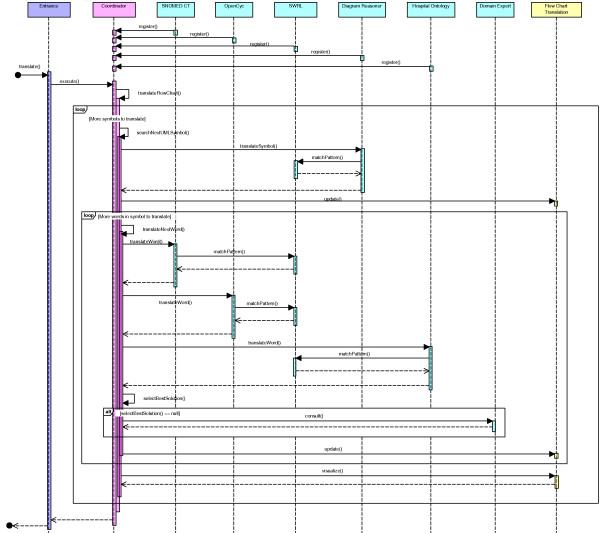
**The sequence diagram of the Combined Reasoning (CR) module**. Represents how all the components of the *Combined Reasoning module *interact with each other and how the data flows between them. The same colors for the classes are used as in Figure 4. Note that all the function calls are synchronous, which means that the next action cannot be started before the previous one is ended.

The complete workflow of the *Combined Reasoning module *is depicted in Figure 5. The *Blackboard *thus contains the flow chart in different stages of transformation. The *Coordinator *investigates which of the *Knowledge Sources*, namely the ontologies with accompanying SWRL Rules and the Diagram Reasoner, can contribute to the solution at this point. The *Coordinator *also determines which *Knowledge Source *will offer a partial solution which brings us the closest to the final solution, namely the complete translation. This *Knowledge Source *is allowed to execute and write this partial solution on the blackboard. When the translation process gets stuck, questions are posed to the domain expert until the issue is resolved. Incorrect translations are detected by the domain expert who monitors the translation. After each step of the translation process, the Drools Rule Flow which has been constructed so far is shown to the domain expert. As can be seen in Figure 3 it looks a lot like the original flow chart and is thus easy interpretable by the domain expert. If the expert detects an error in the flow chart or if additional exceptional situations need to be supported, he or she can correct it before continuing the process by using an intuitive Rule Editor GUI, which allows to add Rules to the application. The expert can also attach an explanation or remarks. These new Rules can be represented in a user-friendly GUI to the domain expert. In case it is not what he/she had in mind, it can quickly be adapted, without loss of development time. This also decreases the evaluation time as the application does not have to be completely deployed before errors are noticed. At the end of the translation the whole Rule Flow is visualized to the domain expert. It is now ready for initial evaluation. The human-readable flow can be evaluated by various medical staff members and corrections can be made through the GUI. As a last step an extensive clinical evaluation has to take place to make sure that the application makes the appropriate decisions under the various possible circumstances.

### Integration into a service-oriented platform

As mentioned in the Related Work section, this architecture was integrated into a Service-Oriented Architecture (SOA), namely the *ICSP platform*.

**Figure 6:**
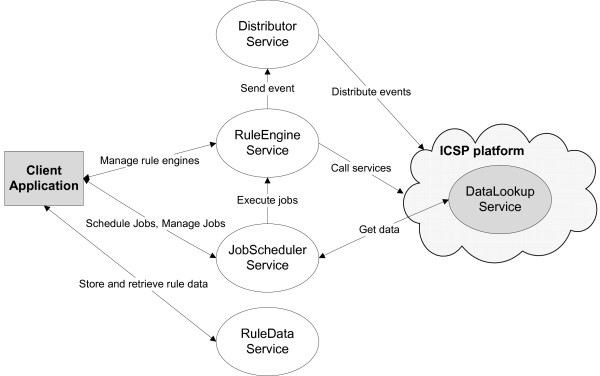
**Interaction view of the integration framework**. Represents how all the components of the integration framework interact with each other and how the data flows between them. The modules in white represent the *Rule Engine Service *and its utilitary services. The gray modules depict a random *Client Application *which uses the Rule-based system and the *ICSP components*. The *DataLookupService *allows the ICSP platform to retrieve data from a database. The *RuleDataService *is used as a persistent container for reusable model data. The *RuleEngineService *offers the actual Rule-based functionality. The *JobSchedulerService *allows to automatically retrieve data from a database. The *DistributorService *an event handling service which provides access to the existing services in the *ICSP platform*.

A general applicable framework was developed that integrates a Rule-based system within an existing SOA, as visualized in Figure 6. The gray modules depict a random *Client Application *which uses the Rule-based system, e.g. a GUI or an ICSP service, and the *ICSP components*. The *DataLookupService *[72] is a Web Service which allows the *ICSP platform *to retrieve data from a database in a flexible manner. The modules in white represent the *Rule Engine Service*, which contains the Rule Flows about the clinical guideline, and its utilitary services. The *Rule Engine Service *offers the actual Rule-based functionality. It contains methods for maintaining the various *Rule Engine *instances, adding and removing Rules and Facts to a certain *Rule Engine *instance and firing the Rules which initiates the process of matching the Facts on the Rules. It is fully integrated into the SOA as a Web Service [73]. The *RuleDataService *is used as a persistent container for reusable model data such as Rules and Facts.

This platform is able to support different *Rule Engines *and even different instances of the same *Rule Engine *at the same time. This allows every application to use a separate instance with its own Rule set and Fact base. To integrate a new *Rule Engine*, the developer only needs to write a new *Adapter module *that translates the generic Rule format used within the platform to the specific Rule language constructs of the *Rule Engine*. Currently, only an *Adapter *for Drools is written.

The framework supports a wide variety of applications by providing the *Rule Engine *instances with generic input and output options for maximum compatibility with the existing services. The *Rule Engine Service *can receive and retrieve input from any arbitrary source and can send output to every future and current service.

To achieve a generic input mechanism the user can decide what the incoming data model (Facts) looks like and can then define Rules using this model. To allow this while still remaining compatible with various external services, a module was developed that can automatically translate the dynamic model of the Facts into Java Beans. The fields of these beans however differ from Fact to Fact. This makes it impossible to construct a generic representation of a Fact based on for example an interface. To resolve this, the program analyzes the data model specified by the user and dynamically generates the required beans at runtime [74]. ASM [75], a framework for Java byte code manipulation, was used to support this functionality. It is possible to process Facts that are delivered directly by a service, but the possibility should also exist to collect the data automatically from the database. However, the *Rule Engine Service *is a passive component that does not take initiative and only processes requests from client applications. To support the automatic retrieval of data the *JobSchedulerService *was implemented which provides the possibility to define tasks (jobs) and plan them for execution at a certain time. During the execution of a job, data (Facts) is retrieved from a component, e.g. the *DataLookupService*, and transformed to input for the *Rule Engine Service*.

The output of the *Rule Engine Service *consists of Actions which can be executed as the consequence of a Rule. This generic output mechanism was achieved by providing a fully generic *Action Descriptor Class *and a processing engine that interprets and executes these descriptors. New output possibilities can be added by extending the *Action Processing Engine*. Currently available actions are the distribution of events to the *DistributorService *and the execution of an arbitrary Web Service method. This last action is easily supported by executing SOAP [76] requests that can intuitively be created using a GUI. The *DistributorService *is an event handling service which serves as the connection point to the existing *ICSP platform*. It allows services from the *ICSP platform *to subscribe to events they are interested in. A Rule can have as consequent a service call to the *DistributorService *to trigger a certain event. The *DistributorService *then alerts all the services which are interested in this event. The publish/subscribe mechanism of the *DistributorService *was implemented through a technology called SAVAN [77], a C implementation of the WS-Eventing specification.

To ease the integration of this program into other applications, all the data, such as the Rules, are saved in an XML-format by using XStream [78].

### Evaluation methods

This subsection details the methods which were used to evaluate the performance of Drools and the proposed architecture, namely the BMI application and the clinical ICU use case, the sedation guideline.

#### Drools performance evaluation

To get a better understanding of the execution times of Drools, we used the following test case. An application categorizing Body Mass Index (BMI) values was developed as a Java class, a Drools Rule file, a Drools Rule Flow and a BPEL application.

The Drools Rule Flow to categorize BMI values was also used to analyze the loading times of the *Production memory *and the *Working memory *of the Drools production system. A *Logger *is also loaded when the application is started. The loading of the *Production memory *consists of loading the Rule Flow and the Rule files, constructing the Knowledge Base and the packages and checking for faults. The influence of the number of decision nodes in the Rule Flow on the loading time was also examined. This was done by constructing a Rule Flow and each time increasing the number of *Split *nodes. The result is comparable to a binary search tree. This way Rule Flows with 1, 3, 7, 15, 19, 24, 28 and 31 *Split *nodes were constructed.

All tests were executed on a MacBook Pro (2.4 GHz Intel Core 2 Duo, 2 GB RAM, Mac OS X version 10.5.7) using the IDE Eclipse 3.4.2 with the Drools plug-in. The BPEL application was executed in NetBeans 6.5.1 with a GlassFish V2 application server. Each test was repeated 10,000 times.

#### ICU use case: sedation guideline

The sedation guideline [79] is a clinical guideline, used for managing the depression of consciousness of critically ill patients in case of severe trauma or for patients needing ventilatory support due to respiratory, cardiac or neurologic failure.

The choice of sedative agents differs according to the expected duration of the sedation, and the given doses will vary depending on the desired sedation level of the patient (light sedation vs. deep comatose patients). These choices are translated into three different sedation guidelines, i.e. the short, normal and deep/long-term sedation guideline. Patients requiring only a short term of sedation are given the most quickly and short acting medication, by which the required sedation level is easily achieved. The other patients will be given less expensive, but longer acting and more difficult to titrate medications. The sedation level of the patient is measured by the Richmond Agitation-Sedation Scale (RASS) [80], which has values ranging from -5 to 4, with -5 being maximal depression of consciousness, and 0 being fully awake and calm. Values above zero mean that the patient is somewhat anxious (value 1), tries to remove tubes or equipment (value 2 and 3) or is violent (value 4).

The main difficulties of the automatic follow-up process of this guideline are:

• The medical staff has to decide whether short or long acting sedative medication will be used, which will be reflected in the choice of sedation guideline (short vs. normal vs. deep/long-term sedation guideline). When patients are kept too long on the short acting medication, the costs become very high. Additionally, the duration of mechanical ventilation (MV) has previously been linked to an increased risk of developing Ventilator-Associated Pneumonia (VAP) [81]. VAP is one of the more frequently encountered nosocomial infections in the ICU [82]. Reducing the duration of MV and length of stay in the ICU are therefore important issues in the ICU setting. Recent studies have shown that the use of a sedation algorithm to promote tolerance to the intensive care environment and preserve consciousness resulted in a marked decrease in the duration of MV and VAP [83].

• A goal RASS, which indicates the sedation depth that needs to be achieved, has to be determined at the beginning of the sedation by the physician. This is sometimes not clearly communicated between the various staff members who care for the patient.

• The RASS score needs to be registered by the nursing staff every four hours, but this is not always strictly followed due to the high workload.

• The correct RASS score needs to be achieved and the proper actions need to be taken to lower or elevate the sedation level. If the patient is sedated too deep or too long with long acting drugs, it will take longer to wake him or her up, which will lead to an increased duration of ventilation and length of stay in the ICU, all causing an increase in costs.

• All the medication has to be entered in the medical database.

To execute this guideline, the medical staff currently uses 5 paper flow charts, which can be found in Additional file 3. These flow charts contain many ambiguities and simplifications and do not enforce correct and timely execution of the guideline. An application, using Drools Rule Flow, was developed to facilitate the use of the guideline and mediate the previously mentioned problems regarding the follow-up of the guideline. To evaluate this application, the same execution environment was used as for the Drools performance tests.

## Results

This section further investigates the suitability of the Drools Rule language for the implementation of clinical guidelines by exploring its performance. The translation of the clinical use case, the sedation guideline, into a Drools Rule Flow application is also presented.

### Drools performance results

**Figure 7:**
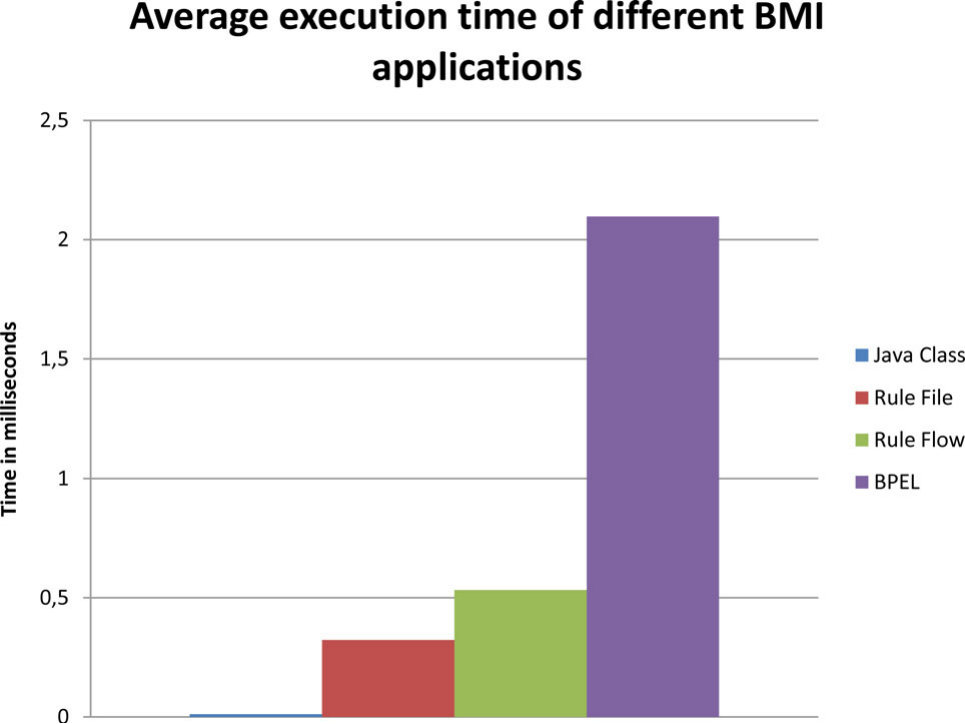
**Average execution time of the different implementations of the BMI application**.

The execution times of the various BMI applications are shown in Figure 7. The standard deviations are respectively 0.0004, 0.0011, 0.0038 and 0.0195 ms. The plain Java class outperformed the other applications. The reason is the overhead generated by the other applications. The execution of a Rule-based system introduces the processing and loading of external files. The BPEL application needs to make a Web Service call to the application server. However, a Java class is much less comprehensible and clear then a Rule (Flow) file. It would be very difficult and error-prone to implement a complicated clinical guideline in Java as this would lead to a lot of intertwined if-structures. However, the execution times of all the applications are below 2 ms, which is negligible.

**Figure 8:**
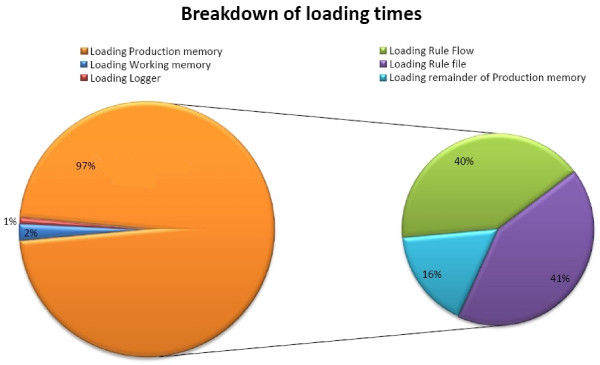
**Analysis of loading times of the working and production memory of a Rule Flow application**.

The results of analyzing the loading times of the *Production memory *and *Working memory *can be seen in Figure 8. The *Production memory *occupies 97% of the loading time, whereas the *Working memory *(Facts) only takes 2%. The remaining time (1%) is used by the loading the *Logger *of the program. Loading the BMI Rule Flow takes approximately the same time as loading the BMI Rule file. The total loading time of the application was on average 2500 ms.

These results can be explained by noting that in a Rete-based production system, the most loading time is consumed by building the Rete network. Here the *Working memory *(the data) is loaded first and then the *Production memory *(the Rules). The Rete network can only be build, when both the Facts and Rules are loaded. This makes it seem like loading the *Production memory *takes a lot of time. If we would load the *Production memory *first and then the *Working memory*, it would seem that loading the data takes a lot of time.

**Figure 9:**
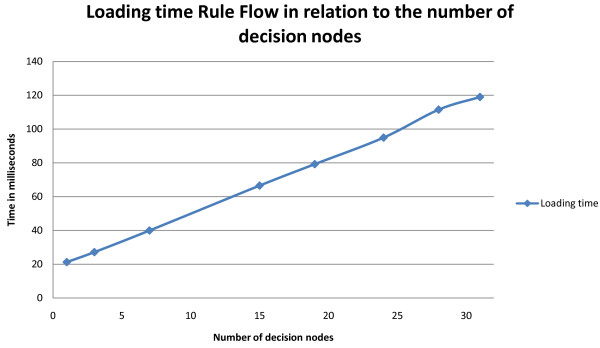
**Loading time of a Rule Flow as a function of the number of decision nodes**.

Finally, the influence of the number of decision nodes in the Rule Flow on the loading time is visualized in Figure 9. As can be seen, the loading of a Rule Flow is linear in relation to the number of decision nodes in the file. The standard deviations are respectively 0.2708, 0.5254, 0.4039, 0.4159, 0.7278, 0.8432, 0.5039 and 0.4905 ms.

We can conclude that the overhead of using Rule Flows will mainly be introduced by the initial loading time which is needed to build the Rete network. However, this only has to be executed once when the application is started.

### Translation of the sedation guideline

**Figure 10:**
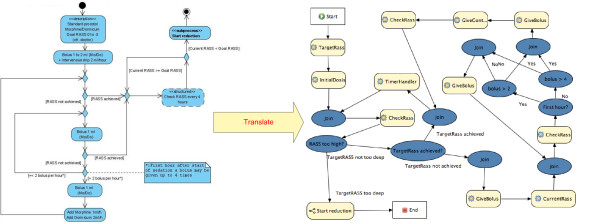
**Manually constructed UML and Drools Rule Flow of the sedation guideline**.

To explore the difficulties the semi-automatic translation could run into, the paper flow charts of the sedation guideline (see Evaluation Methods subsection) were manually translated by using the same resources and methods as the platform would. The original, manually constructed flow chart (UML diagram) of the normal sedation guideline and the Drools Rule Flow it was translated to, can be viewed in Figure 10. As can be seen, this flow chart contains some simplifications, e.g. modeling an exceptional situation as a footnote instead of as a part of the flow chart, and ambiguities, e.g. not indicating how much time should be left between adjusting the medication and checking the RASS again.

**Figure 11:**
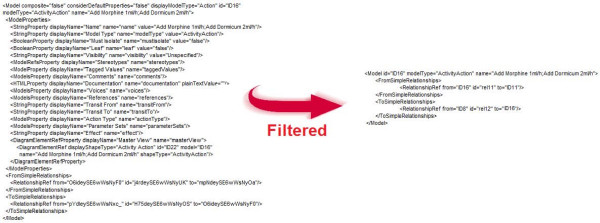
**Example of XML filtered by the Pre-processing Module**.

First the XML of the original UML is filtered by the *Pre-processing module*. As visualized in Figure 11, all the graphical information is filtered. The important information is the type of the model symbol used and the information about the connections of this model element to the other model elements. Next, this filtered XML is translated to the intermediate language by the *Combined Reasoning module*. This intermediate language is later translated to Drools Rule Flow by the *Post-processing module*. The method *searchNextUMLSymbol() *reads a word from the XML-file until it encounters the <*Model *construct. This indicates that the start of the description of a symbol in the UML diagram is reached. The method then searches for the *id, type *and *name *of the symbol by looking for constructs of the form "*id=12*", "*modelType=ActivityAction*" and "*name=Add Morphine 1ml/h, Add Dormicum 2ml/h*", as illustrated in Figure 11.

**Figure 12:**
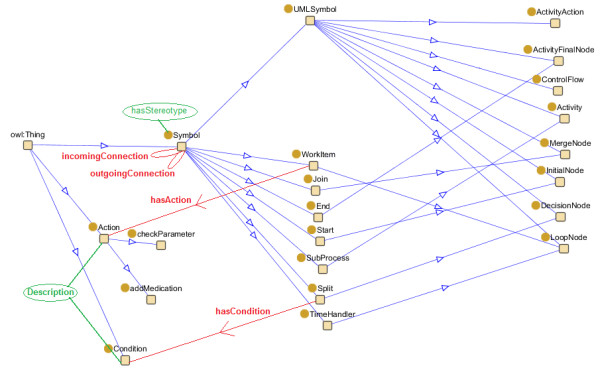
**The Diagram Ontology**. Represents a part of the Diagram Ontology. The yellow squares represent the classes. Blue arrows indicate subclass relationships. Red arrows and lines indicate relations between classes (object properties). Green Ovals represent attributes of classes (datatype properties).

The symbol is translated by the *Diagram Reasoner Knowledge Source *to the correct construct in the intermediate language by using the *Diagram ontology*. A part of this ontology is visualized in Figure 12. The *modelType *is matched with the names of the subclasses of the *UMLSymbol *OWL class in the ontology. A new instance is created in the ontology of the appropriate OWL class, with as name the *id *of the symbol. By reasoning on this ontology instance with Pellet, it is discovered which intermediate language symbol(s) should be created. By inspecting the properties of the OWL classes that represent these intermediate symbols, it is also known which additional XML-properties of the symbol should be translated. For example, if we want to translate a symbol with *modelType InitialNode *and *id ID34*, an instance of the *InitialNode *OWL class is created in the ontology with name *ID34*. Pellet reasons that a *Start *symbol needs to be created in the intermediate language. The *Start *OWL class needs an *outgoing connection *and can have no *incoming connections*.

However, most of the time the decision of which symbol(s) should be created is not so straight-forward and cannot be expressed by utilizing OWL DL. In these cases, SWRL is used. For example, if we want to translate the filtered XML from Figure 11, an instance of the *ActivityAction *OWL class is created in the ontology with name *ID16*. However, symbols of the type *ActivityAction *can be used for various purposes. A *Stereotype *property can be defined in the XML that gives an indication for which purpose the symbol is used such as "*description*" or ''*subprocess*" or "*wait*". The value of this property is attached to the *ActivityAction *OWL class in the ontology through the *hasStereoType *property. The following SWRL Rules are defined:

ActivityAction(?x) ⋀ hasStereoType(?x, ?y) ⋀ swrlb:stringEqualIgnoreCase(?y, "wait") → TimeHandler(?x)

ActivityAction(?x) ⋀ hasStereoType(?x, ?y) ⋀ swrlb:stringEqualIgnoreCase(?y, "subprocess") → SubProcess(?x)

ActivityAction(?x) ⋀ (hasStereoType = 0)(?x) → WorkItem(?x)

It is concluded that a *WorkItem *needs to be created in the intermediate language. The *WorkItem *OWL class needs an associated *Action *and an *incoming *and *outgoing Connection*.

The *Diagram Reasoner *creates the *Action *OWL class out of the *name *property which was previously read. This *name *needs to be analyzed by pattern matching the different words in this *name *property to the names of the classes in the various ontologies such as OpenCyc, SNOMED CT and LinkBase. An ontology also exists that models all the knowledge that is available in the database of the ICU of the hospital. This ontology can be mapped on the database by using D2R [84]. Not all classes can be mapped on information in the database. SWRL Rules can be defined on top of the ontology to indicate how this missing information can be calculated, e.g.:

Patient(?x) ⋀ hasWeight(?x, ?y) ⋀ hasHeight(?x, ?z) ⋀ swrlb:multiply(?k, ?z, ?z) ⋀ swrlb:divide(?bmi, ?y, ?k) → hasBMI(?x, ?bmi)

These SWRL Rules can then in turn be analyzed by the translator to know how the parameter should be calculated in the eventual Drools Rule Flow.

If we want to translate the action "*Add Morphine 1ml/h, Add Dormicum 2ml/h*", the word *Morphine *can be matched to the names of the OWL classes in the *Hospital ontology*. A *Morphine *OWL class, which is a subclass of the *Medication *OWL class, is discovered. We need to discover a leaf node in this ontology as we need to find the specific medication a patient needs. The ontology contains various OWL subclasses of *Morphine*. The program tries to differ amongst these different possibilities by involving other words specified in the action such as *1ml/h and 2ml/h*. It still arrives at two possible solutions:

• Morphine IV [20mg/1ml]

• Morphine IV [10mg/1ml]

The *selectBestSolution() *method specifies Rules (not in SWRL) that can be used to differ amongst the various solutions e.g. proximity of *1ml/h *to the *Morphine *word. Such Rules can also be used to choose between different solutions which are given by the various ontologies. If the Rules still do not offer a solution, these options are shown to the domain expert in a user-friendly GUI. He/she can indicate which medication is correct. As a consequence a new instance of the *Medication *OWL class is created in the ontology.

The same process is followed to discover the correct *Dormicum *medication. However, we still need to determine which type of *Action *we need to create. Therefore, we check if there is already a type of *Action *that complies with the information we have already found. By doing pattern recognition we discover that the *AddMedication *OWL class in the ontology meets our needs. An instance of this OWL class is created with the discovered *Medication *individuals attached to it.

Only the connections need to be created now. Therefore, the *Diagram Reasoner *uses the *translateNextWord() *method until it reaches the <*RelationshipRef *construct and analyzes its *From *and *To *properties to create the appropriate relations in the ontology. If the symbol to which this symbol needs to be connected does not exist in the ontology yet, it is created as an instance of the high-level OWL class *Symbol*. This symbol is then defined more specifically at a later stage of the translation. 

At this point the symbol is completely translated. All the information, which was created in the ontology (the instances), is translated to the intermediate XML. The *Post-processing *module then translates the intermediate XML to Drools Rule Flow XML and creates the necessary Java classes. This intermediate result can be visualized to the domain expert for initial evaluation. He/she can intervene at this point if an error has occurred in the previous translation step.

Finally, the next symbol can be translated. This whole process is repeated until the end of the filtered XML document is reached. This ends the translation process. At this point the complete result is visualized to the domain expert, who can make changes if needed.

The problem could occur that the symbol cannot be found in the *Diagram Ontology *e.g. the symbol with *ModelType Note*, visualized by the asterisk at the bottom of the original UML figure. There is no general definition of how this symbol should be handled. The content of the *name *property is analyzed with the ontologies as was done in the above example and the results are visualized to the domain expert. He/she can indicate which type of symbol should be created in the *Diagram ontology*. Additional questions will be asked by the *Diagram Reasoner *to the domain expert to fill in the properties of this symbol. The domain expert can also choose to model this part of the Drools Rule Flow him/herself from scratch by using the intuitive GUI.

Similarly, it could occur that a *name *property cannot be translated because the ontologies do not contain enough information about this subject. The property is presented to the domain expert who can take various actions. He/she can correct spelling errors or provide an alternate formulation and try the translation again. If this does not work, he/she can opt to add a new definition to the ontology. The domain expert can select the word out of the *name *property for which he/she wants to add a definition to the ontology. A high-level overview of the ontology is visualized to the domain expert who can then select the best high-level concept that represents this word. The program then leads the domain expert further down the tree until a leaf node is reached or no appropriate classes are available. At this point a new class can be created e.g. as an equal class (synonyms) or a subclass. An indicator is added to this class to alert the ontology engineer of this change. Finally, if all these options do not offer a solution, the domain expert can opt to model this part of the Drools Rule Flow him/herself with the GUI.

As can be seen from the previous examples, a domain expert always needs to be involved in the translation process as it is difficult for a computer to interpret the ambiguities and simplifications in the guideline.

### The sedation Rule-based application

**Figure 13:**
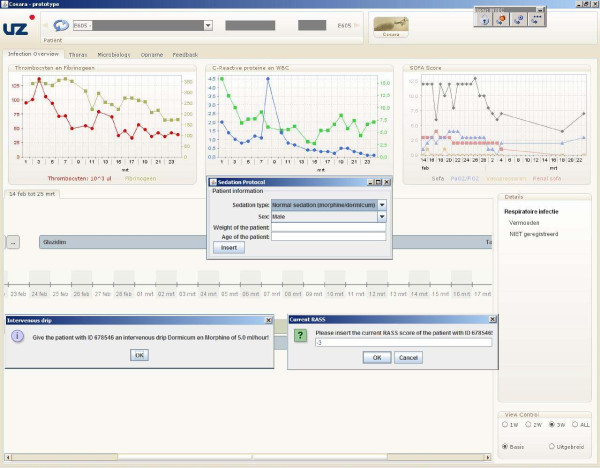
**GUI of the sedation application**. The Graphical User interface (GUI) which allows nurses to easily interact with the sedation application and input the needed information about the patient, the RASS score and the medication. The "Input patient information" screen is shown at the start of the application. It allows the nurses to input information about the patient (weight, age and sex) and which type of sedation (short vs. normal vs. deep/long-term) guideline should be used. If this information is already available in the database, the fields will be filled in with the data found in the database. The nurse can then adjust or confirm this data. The "Medication notification" screen is an example of a message that is displayed on screen when the medication of the patient should be adjusted. The "Input current Rass" screen prompts the nurses to input the current RASS of the patient. The time points, at which these RASS scores should be collected, are prescribed by the sedation guidelines. For example, in the normal sedation guideline, the RASS score needs to be updated every four hours. This means that this screen will automatically be shown every four hours, unless the RASS score has already been inputted in the database through another interface.

The translation of the 5 flow charts results in 7 Drools Rule Flows. First, a Rule Flow of 28 nodes for the normal sedation guideline and two Rule Flows of 32 and 28 nodes for the short sedation guideline. The Rule Flow that encodes reducing the normal sedation contains 22 nodes and the Rule Flow for reducing the short sedation contains 30 nodes. Two Rule Flows were created to combine these various Rule Flows. They contain 5 and 4 nodes. This adds up to 149 nodes to encode the complete sedation protocol. The developed sedation application will give the nursing staff notifications on steps to be taken and will increase the usability of the guideline. It was also easily integrated in the already available ICSP platform by employing the integration framework as discussed in the section "Integration into a service-oriented platform". A GUI, visualized in Figure 13 was additionally developed to allow the nurse to easily interact with the application and input the needed information about the RASS score and medication.

**Figure 14:**
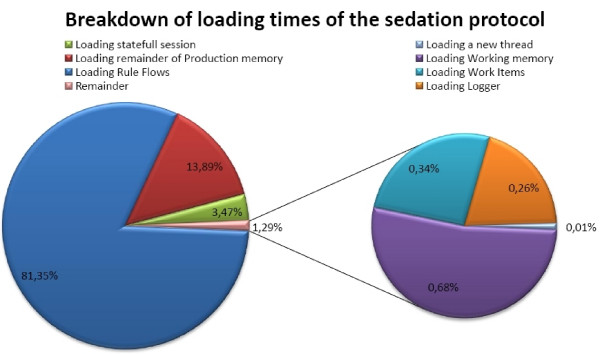
**Analysis of the loading times of the sedation guideline Drools Rule Flow**.

To get a better insight into the internal workings of sedation guideline application, the different loading times were analyzed as illustrated in Figure 14. It is obvious that loading and parsing the Rule Flow files consumes the most time. To reduce these loading times as much as possible, it was opted to only load those Rule Flows that were currently necessary to execute the guideline. Thus, if the patient is currently sedated according to the normal guideline, only the Rule Flow of this type is loaded. The disadvantage is that these files can only be loaded after the type of sedation is determined. This means that the nursing staff will have to wait a couple of seconds after inputting this information before they get the first instructions of the guideline. The average loading time of a Drools Rule Flow of the sedation guideline is 2719 ms. After the loading of the Rule Flows, the loading of the rest of the *Production memory *consumes the most time. This mainly includes the initialization of the different Java classes.

## Discussion

The results in the previous section indicate that Drools is an effective Rule language for implementing clinical guidelines. It has a good performance by utilizing a object-oriented implementation of the Rete algorithm. This algorithm sacrifices memory for speed. However, most of the memory usage was consumed by loading the Rule Flows. This only has to be done one time, namely when the application is started. The memory usage of the applications does tend to increase as the number of Rules and/or Facts increases. This is caused by the Rete algorithm as it attaches the Facts in memory to the nodes in the Rules Tree with which they match. This can easily be countered by keeping the Rule Flows simple by splitting large flows up into smaller ones as we have done in the sedation application.

The main weakness of our performance study is that we only evaluated the performance of Drools based on two use cases namely calculating BMI and the sedation guideline. This makes it difficult to generalize the results outside the context of these two examples. However, the performance of Drools has been evaluated on numerous benchmarks [85]. These benchmarks do not contain medical data and the evaluation mostly focuses on comparing different *Rule Engines *(or different versions of Drools). Our case study compares Drools with other technologies such as BPEL and focuses on medical data. Moreover, by translating a specific medical use case we gained a lot of insight into the strengths and weaknesses of the platform and the shortcomings of the used ontologies.

Drools is well-suited to support the semi-automatic translation of XML-based flow charts into working programs as the Drools Rule Flows closely resemble the original flow charts and have an XML-based encoding. Drools also allows for quick development, evaluation and visualization of the Rules by providing various user-friendly GUIs and an integration with the Eclipse IDE. Although, the flow charts will be automatically translated, this can still be very useful. At the end of the translation process, the Drools Rule Flow can be visually represented in tree-based manner to the medical staff for example a physician. This physician can then quickly perform a first evaluation if the clinical guideline was correctly translated and implemented.

Semi-automatically translating the flow charts into Rule Flows, leads to less development and evaluation time. The IT developer does not have to be present for each guideline that needs to be implemented. An XML-based flow chart, which can easily be constructed by various graphical flow chart editors such as Visual Paradigm [86], can be given to the application by a physician. The application automatically translates the guideline to a Rule Flow. Whenever it runs into a problem, natural language questions are posed to the physician until the translation process can move on. This also detects and resolves ambiguities that were present in the original flow chart.

In the Introduction some problems in manually constructed guidelines were identified which make it difficult to computerize these guidelines. These problems are addressed in our translation framework. The lack of definitions is solved by using ontologies, such as OpenCyc, WordNet, LinkBase and SNOMED CT. Ontologies can exactly be used to store formal knowledge such as medical terms (e.g. synonyms) and English constructs (e.g. IF...THEN). The domain expert intervenes when incomplete information, ambiguities and simplifications in the guideline become apparent during the translation. To verify if the flow charts are complete, it is checked that each if construct is also accompanied by an else construct. If not all situations are covered in the guideline, this will also become apparent during the clinical evaluation of the translated guideline. This guideline can then easily be adapted by inserting new Rules with the user-friendly GUI. The domain expert also specifies the necessary parameters. Because flow chart guidelines are translated, the correct level of abstraction is used, the events can be timed and no omission errors can occur. The flow charts that are translated are suitable for execution as they are already used in the ICU of the Ghent University hospital.

As mentioned previously, the physician can evaluate the outcome visually before running it on some test cases. The IT developer can support the physician, when the guideline is not correctly implemented by the translation process. He or she can use the Rule editor GUI, as described in the section "Semi-automatic translation", to adapt the Rule Flow and visualize the result to the physician.

A computer-based clinical guideline leads to an improved execution of the clinical guideline. It reminds the medical staff of tasks that need to be performed by giving notifications. This ensures a more timely and correct execution of the guideline. The Rule-based system is capable of logging the Rules that were fired to reach a specific medical recommendation. This will allow medical staff members to check which Rules have been fired and how a certain conclusion was reached. This will increase the trust of the staff members in the application. We expect that this will also increase the compliance of the staff members, although this still needs to be evaluated empirically. Exceptional situations and problems, e.g. a patient that has been sedated too long or too deep, can easily be detected by the application as it continuously monitors the parameters of the patient. The application can also give the medical staff a notification when some information is missing, e.g. the goal RASS or the current medication of the patient is not entered into the database. Finally, the condition of the patient and previously made decisions can be nicely visualized. This helps to quickly follow the condition of the patient and supports the communication between the different staff members.

The same framework could be used to implement guidelines for therapy of more chronic diseases (e.g. diabetes type 2 or hypertension). The main difference is however the fact that the intensive care unit is extremely data-rich and a lot of the necessary data can be automatically obtained from linking to GLIMS (global laboratory information management system) and the intensive care information system or a computerized physician order entry system (CPOE). Furthermore, this sedation guideline requires a lot of staff intervention and need for continuously monitoring of data. Because the computerized decision support system automatically and continuously monitors the data and gives suggestions to the staff accordingly, a lot of benefit is offered to the medical staff. However, for chronic disease guidelines, the time between interventions is larger and it is not possible to monitor data fully automatically. The nature of guidelines is different, and the workflow-driven process framework is not as effective as has been illustrated by Tu et al. [26] with the EON framework.

As an extension, a self-learning component, which further improves the execution and follow-up of clinical guidelines, could easily be integrated into the infrastructure. On the one hand, many guidelines do not provide recommendations for all clinical situations encountered in practice. These situations could easily be detected by the program either because there is no Rule available that handles the situation or a wrong Rule is executed and the medical staff member will choose not to follow the recommendations. On the other hand, medical staff members sometimes choose not to comply with the clinical guidelines and the given recommendations. These situations and the decision that were consequently made by the staff members could then easily be investigated by various data mining techniques [87]. The feedback of these studies could in turn be used to optimize the clincal guideline.

Future work will focus mainly on evaluating if the semi-automatic translation of guidelines is more efficient than manually encoding them. Therefore the semi-automatic translation architecture will be applied to various clinical guidelines in different areas of ICU decision support. These guidelines will also be manually translated. In both cases, the time it took to completely and correctly translate and evaluate the guideline, the amount of human effort needed and the number of errors still present in the computerized guideline will be measured and compared to each other. Simultaneously it will be evaluated if the current ontologies and SWRL-Rules are generally applicable to this wide range of use cases and how much the domain expert is needed to support the translation process.

Note that the evaluation needs to be applied to multiple guidelines as the semi-automatic translation framework will perform better after a couple of translations. The most common problems will be resolved by the domain expert during the first couple of translations and these solutions will be stored in the various ontologies and Rules.

It is important to do a very thorough evaluation of the translated guidelines in a clinical setting involving medical staff and real patients.

## Conclusions

In this article a semi-automatic verification and translation framework, capable of turning manually constructed XML-based flow charts into ready to use Drools Rule Flow programs is proposed. For this, semantic technologies, such as ontologies and SWRL, are used. This framework aims to close the gap in communication between the medical staff and the computer scientists for the construction of computer-based clinical guidelines. This closer collaboration results in a less time-consuming and less error-prone development phase and a shorter clinical evaluation phase. 

Drools was shown to be an effective Rule language for computerizing clinical guidelines by translating the sedation protocol to a Drools Rule Flow. Drools allows for quick development and evaluation, human-readable visualization of the Rules and has a good performance.

The computer-based clinical guideline supports an improved execution of the clinical guideline by giving the medical staff reminders and notifications of tasks that need to be performed and by automatically detecting exceptional situations and problems by monitoring the parameters of the patient. Future work will focus mainly on evaluating if the semi-automatic translation of guidelines is more efficient than manually encoding them by applying the proposed architecture to various clinical guidelines in different areas of ICU decision support.

## List of abbreviations used

ADD: Attribute Driven Design; AI: Artificial Intelligence; BMI: Body Mass Index; BPEL: Business Process Execution Language; CDSS: Computerized decision support systems; CfMS: Care flow Management System; CPOE: Computerized Physician Order Entry system; CR module: Combined Reasoning module; EHR: Electronic Health Record; EPR: Electronic Patient Record; FMA: Foundational Model of Anatomy; Galen: Generalized Architecture for Languages, Encyclopaedias and Nomenclatures; GIMS: GuIdeline Management System; GLIF: GuideLine Interchange Format; GLIMS: Global Laboratory Information Management System; GO: Gene Ontology; GUI: Graphical User Interface; HL7: Health Level 7; ICSP: Intensive Care Service Platform; ICT: Information and Communication Technology; ICU: Intensive Care Unit; IDE: Integrated Development Environment; IFOMIS: Institute for Formal Ontology and Medical Information Science; IT: Information Technology; IZIS: Intensive Care Information System; Jess: Java Expert System Shell; L&C: Language and Computing; MKM: Medical Knowledge Modules; MTBA: Modeling Better Treatment Advice; MV: Mechanical Ventilation; NCI: National Cancer Institute; NLP: Natural Language Processing; OBI: Ontology for Biomedical Investigations; OBO: Open Biomedical Ontologies; OWL: Ontology Web Language; PC: Personal Computer; PDA: Personal Digital Assistant; RASS: Richmond Agitation-Sedation Scale; RIM: Reference Information Model; SNOMED CT: Systematized Nomenclature of Medicine-Clinical Terms; SOA: Service-Oriented Architecture; SWRL: Semantic Web Rule Language; UML: Unified Modeling Language; VAP: Ventilator-associated pneumonia; WfMS: Workflow Management System; XML: eXtensible Markup Language.

## Competing interests

The authors declare that they have no competing interests.

## Authors' contributions

FO, FDB and KS carried out the study, participated in the development of the concepts described in this paper and drafted the manuscript. KC participated in the case study. WK participated in the development of the integration framework. FDT and JD supervised the study, participated in its design and coordination and helped to draft the manuscript. All authors read and approved the final manuscript.

## Pre-publication history

The pre-publication history for this paper can be accessed here:

http://www.biomedcentral.com/1472-6947/10/3/prepub

## Supplementary Material

Additional file 1 **Appendix A: Overview of the most prevalent formats for representing clinical guidelines **The pdf (appendixA.pdf) contains a description of the standardization efforts and formalisms for representing clinical guidelines that have been proposed in literature, namely the Arden Syntax, PROforma, EON, GLIF, PRODIGY, Asbru and Guide. Click here for file

Additional file 2 **Appendix B: Overview of the existing medical and natural language ontologies which can be used to support the translation process **The pdf (appendixB.pdf) contains an overview of the existing medical and natural language ontologies that can be (partially) reused to support the semi-automatic translation process. Cyc and WordNet are two well-known ontologies that model general knowledge about the English language. A wide range of ontologies about the eHealth domain are described such as LinkBase, SNOMED CT, the Galen Common Reference Model, the NCI Cancer Ontology, the Foundational Model of Anatomy Ontology (FMA), the Gene Ontology (GO) and the Ontology for Biomedical Investigations (OBI). All these ontologies are available in OWL. Click here for file

Additional file 3 **The 5 flow charts (UML diagrams) of the sedation guideline **The zip (sedationGuidelines.zip) contains the 5 flow charts (UML diagrams) of the sedation guideline in pdf format. Click here for file
