# Supplementary material for: Towards computerizing intensive care sedation guidelines: design of a rule-based architecture for automated execution of clinical guidelines
Source: BMC Med Inform Decis Mak. 2010 Jan 18;10:3. doi: 10.1186/1472-6947-10-3 (PMC2823596; doi:10.1186/1472-6947-10-3)
Supplement: Additional file 1 — Appendix A: Overview of the most prevalent formats for representing clinical guidelines The pdf (appendixA.pdf) contains a description of the standardization efforts and formalisms for representing clinical guidelines that have been proposed in literature, namely the Arden Syntax, PROforma, EON, GLIF, PRODIGY, Asbru and Guide. [file 1472-6947-10-3-S1.PDF]

Appendix A: overview of the most prevalent formats for representing clinical guidelines

Femke Ongenae*[1], Femke De Backere[1], Kristof Steurbaut[1], Kirsten Colpaert[2], Wannes Kerckhove[1], Johan Decruyenaere[2] and Filip De Turck[1]

[1]Department of Information Technology (INTEC), Ghent University - IBBT, Gaston Crommenlaan 8, Bus 201, 9050 Ghent, Belgium
[2]Department of Intensive Care, Ghent University Hospital, De Pintelaan 185, 9000 Ghent, Belgium

Email: Femke Ongenae*- Femke.Ongenae@intec.ugent.be; Femke De Backere - Femke.DeBackere@intec.ugent.be; Kristof Steurbaut - Kristof.Steurbaut@intec.ugent.be; Kirsten Colpaert - Kirsten.Colpaert@ugent.be; Wannes Kerckhove - Wannes.Kerckhove@intec.ugent.be; Johan Decruyenaere - Johan.Decruyenaere@ugent.be; Filip De Turck - Filip.DeTurck@intec.ugent.be;

*Corresponding author

Representing clinical guidelines

A lot of standardization efforts and formalisms for representing clinical guidelines have been proposed in literature [1–4]. Below an overview is given of the most prevalent formats:

- **The Arden Syntax [5]**: The Arden syntax is a formal, procedural language for representing medical guidelines. The Arden Syntax is adopted as a HL7 (Health Level 7) standard in 1999 [6]. This format uses Medical Knowledge Modules (MLMs), containing the logic necessary for making and implementing one medical decision. An MLM is a hybrid between a production Rule and a procedural formalism. Each MLM is invoked as if it were a single-step IF-THEN Rule, but then it executes a sequence of instructions, including queries, calculations, logic and write statements. The Arden Syntax supports time functions by ensuring that every data element and every event has a timestamp that is clinically significant.

- **PROforma [7, 8]**: PROForma consists of a formal specification language, a knowledge representation language and a set of Prolog and Java tools for building applications in the language. The aim of PROforma is to use a minimal set of constructs to model a guideline. PROforma defines four tasks. Each task has some attributes describing the goal, control flow and conditions for executing the task. An ontology is used to check the soundness.

- **EON [9]**: EON is a component-based suite of models and software components for the creation of guideline-based applications. It was developed in 1996 by Stanford University. EON includes an extensible set of models to represent information in a clinical guideline such as domain ontologies and models of patient data. The guideline model, called the Dharma model, defines the structures necessary to create guideline knowledge. Protégé is used as the environment to create and maintain the models. Patient data is obtained from user input or from a temporal database. Explanations of the given recommendations by the guideline can also be requested.

- **GLIF [10]**: The aim of GLIF was to come to a uniform format, used as an exchange format for guidelines [11]. Therefore, GLIF combines the properties of the Arden Syntax, GEODE-CM (a state-transition framework for clinical management), MTBA (Modeling Better Treatment Advice, a client-server architecture) and EON. GLIF has a formal representation. It defines an ontology for representing guidelines, as well as a medical ontology for representing medical data and concepts. GLIF uses HL7 RIM (Reference Information Model) as data model.

- **PRODIGY [12]**: PRODIGY is a format used to model chronic disease management in primary care, such as asthma, hypertension and angina. PRODIGYs main aim is to create a simple, comprehensible and readily model for representing such guidelines. It supports a series of decisions that a nurse or general practitioner may have to make by enabling a guideline to be organized as a network of patient scenarios, decisions concerning management and actions. HL7 RIM is also used to model patient information.

- **Asbru [13]**: Asbru is a time-oriented guideline format. Asbru enables the intentions and goals of a guideline and the temporal dimensions and uncertainties to be defined as an intrinsic part of that guideline.

- **Guide [14]**: GUIDE consists of 3 main modules. The Guideline Management System (GIMS), the Electronic Patient Record (EPR) and the Workflow Management System (WfMS) (also called the Care flow Management System, CfMS). GIMS provides the clinical decision support, while WfMS takes care of the organizational support. The different modules interact based on messages. These messages are defined by specific contracts. SNOMED CT tags can be used for terminology abstractions.

List of abbreviations used

CfMS: Care flow Management System; EPR: Electronic Patient Record; GIMS: GuIdeline Management System; GLIF: GuideLine Interchange Format; HL7: Health Level 7; MKM: Medical Knowledge Modules; MTBA: Modeling Better Treatment Advice; RIM: Reference Information Model; SNOMED CT: Systematized Nomenclature of Medicine-Clinical Terms; WfMS: Workflow Management System.

Acknowledgements

Femke Ongenae would like to thank the Institute for the Promotion of Innovation by Science and Technology in Flanders (IWT) for her Phd grant.

References

1. De Clercq P, Kaiser K, Hasman A: **Chapter 2: Computer-Interpretable Guideline formalisms**. In *Computer-based medical guidelines and protocols: a primer and current trends*, Amsterdam, The Netherlands: IOS Press 2008:22–43.
2. **Open clinical: Methods and tools for representing computerised clinical guidelines** [http://www.openclinical.org/gmmsummaries.html].
3. Peleg M, Tu S, Bury J, Ciccarese P, Fox J, Greenes RA, Hall R, Johnson PD, Jones N, Kumar A, Miksch S, Quaglini S, Seyfang A, Shortliffe EH, Stefanelli M: **Comparing computer-interpretable guideline models: A case-study approach**. *Journal of the American Medical Informatics Associacation* 2002, **10**:1135–1168.
4. Wang D: **Representation primitives, process models and patient data in computer-interpretable clinical practice guidelines: A literature review of guideline representation models**. *International Journal of Medical Informatics* 2002, **68**(1-3):59–70.
5. Kim S, Choi I: **Arden Syntax as a standard expression language for medical knowledge**. *Journal of Korean Society of Medical Informatics* 2008, **14**:1–7.
6. **The Arden Syntax as HL7 standard** [http://www.hl7.org/implement/standards/ansiapproved.cfm].
7. Sutton DR, Fox J: **The syntax and semantics of the PROforma guideline modeling language**. *Journal of the American Medical Informatics Association* 2003, **10**(5):433–443.
8. Sutton DR, Taylor P, Earle K: **Evaluation of PROforma as a language for implementing medical guidelines in a practical context**. *BMC Medical Informatics and Decision Making* 2006, **6**:20.
9. Tu SW, Musen MA: **A flexible approach to guideline modeling**. In *Proceedings of the American Medical Informatics Association Symposium: 1999; Washington, DC, USA* 1999:420–424.
10. Peleg M, Boxwala A, Ogunyemi O, Zeng Q, Tu S, Lacson R, Bernstam E, Ash N, Mork P, Ohno-Machado L, Shortliffe EH, Greenes RA: **Glif3: the evolution of a guideline representation format**. In *Proceedings of the American Medical Informatics Assocation Annual Symposium: 4-8 November, 2000; Los Angeles, California, USA* 2000:645–649.
11. Ohno-Machado L, Gennari JH, Murphy SN, Jain NL, Tu S, Oliver DE, Pattison-Gordon E, Greenes RA, Shortliffe EH, Barnett GO: **The guideline interchange format: A model for representing guidelines**. *Journal of the American Medical Informatics Association* 1998, **5**(4):357–372.
12. Johnson PD, Tu S, Booth N, Sugden B, Purves IN: **Using scenarios in chronic disease management guidelines for primary care**. In *Proceedings of the American Medical Informatics Association Symposium: 4-8 November, 2000; Los Angeles, California, USA* 2000:389–393.
13. Miksch S, Shahar Y, Johnson P: **Asbru: a task-specific, intention-based, and time-oriented language for representing skeletal plans**. In *7th Workshop on Knowledge Engineering Methods and Languages (KEML-97): 1997; Milton Keynes, UK* 1997.

14. Quaglini S, Stefanelli M, Cavallini A, Micieli G, Fassino C, Mossa C: **Guidelinebased careflow systems**. *Artificial Intelligence in Medicine* 2000, **20**:5–22.
