# Supplementary material for: Towards computerizing intensive care sedation guidelines: design of a rule-based architecture for automated execution of clinical guidelines
Source: BMC Med Inform Decis Mak. 2010 Jan 18;10:3. doi: 10.1186/1472-6947-10-3 (PMC2823596; doi:10.1186/1472-6947-10-3)
Supplement: Additional file 2 — Appendix B: Overview of the existing medical and natural language ontologies which can be used to support the translation process The pdf (appendixB.pdf) contains an overview of the existing medical and natural language ontologies that can be (partially) reused to support the semi-automatic translation process. Cyc and WordNet are two well-known ontologies that model general knowledge about the English language. A wide range of ontologies about the eHealth domain are described such as LinkBase, SNOMED CT, the Galen Common Reference Model, the NCI Cancer Ontology, the Foundational Model of Anatomy Ontology (FMA), the Gene Ontology (GO) and the Ontology for Biomedical Investigations (OBI). All these ontologies are available in OWL. [file 1472-6947-10-3-S2.PDF]

Appendix B: overview of the existing medical and natural language ontologies which can be used to support the translation process

Femke Ongenae*[1], Femke De Backere[1], Kristof Steurbaut[1], Kirsten Colpaert[2], Wannes Kerckhove[1], Johan Decruyenaere[2] and Filip De Turck[1]

[1]Department of Information Technology (INTEC), Ghent University - IBBT, Gaston Crommenlaan 8, Bus 201, 9050 Ghent, Belgium
[2]Department of Intensive Care, Ghent University Hospital, De Pintelaan 185, 9000 Ghent, Belgium

Email: Femke Ongenae*- Femke.Ongenae@intec.ugent.be; Femke De Backere - Femke.DeBackere@intec.ugent.be; Kristof Steurbaut - Kristof.Steurbaut@intec.ugent.be; Kirsten Colpaert - Kirsten.Colpaert@ugent.be; Wannes Kerckhove - Wannes.Kerckhove@intec.ugent.be; Johan Decruyenaere - Johan.Decruyenaere@ugent.be; Filip De Turck - Filip.DeTurck@intec.ugent.be;

*Corresponding author

Medical and natural language ontologies

Existing medical and natural language ontologies can be used to support the translation process. Two well-known ontologies exist that model general knowledge about the English language such as synonyms, generally true statements and so on:

- **Cyc [1]**: Cyc is a knowledge base of everyday common sense knowledge. Cyc enables applications to perform human-like reasoning. In this architecture OpenCyc will be used. OpenCyc is a subset of Cyc, containing over 47,000 terms. OpenCyc is freely available and also available in an OWL version [2].

- **WordNet [3]**: WordNet is a lexical database for the English language. It groups English words into sets of synonyms called synsets, provides short, general definitions, and records the various semantic relations between these synonym sets.

A wide range of ontologies exist about the eHealth domain. Below an overview is given of the most relevant, well-known and well-developed eHealth ontologies, which are available in OWL and that could be (partially) reused to support the semi-automatic translation.

- **LinkBase [4]**: LinkBase is a large, comprehensive and extendable knowledge base which covers various aspects of medicine, including procedures, anatomy, pharmaceuticals and various disorders and anomalies. It contains millions of knowledge elements and is three times larger than SNOMED CT. It is developed by L&C NV (Language and Computing) in close collaboration with IFOMIS (Institute for Formal Ontology and Medical Information Science) of the University of Leipzig. It provides support for extracting discrete data from unstructured or free text and turning that data into information. LinkBase provides a mechanism into which external medical terminologies and databases may be embedded [5].

- **SNOMED CT [6]**: SNOMED CT stands for **S**ystematized **No**menclature of **Med**icine-**C**linical **T**erms. It is a comprehensive medical terminology, containing multilingual clinical healthcare concepts such as diseases, findings and procedures. SNOMED CT improves patient care by facilitating communications and interoperability in electronic health data exchange. SNOMED is a registered standard with the HL7 Vocabulary Technical Committee for use in HL7 messages. SNOMED CT covers a large portion of the medical terms in diagnosis and problem lists [7, 8].

- **Galen Common Reference Model [9, 10]**: Galen stands for **G**eneralized **A**rchitecture for **L**anguages, **E**ncyclopaedias and **N**omenclatures in medicine. The GALEN Common Reference Model is a large ontology of the medical domain centered on significant core content relating to human anatomy. Other content relates to human physiology, pathology and symptomatology as well as pharmacology. The model aims to represent "all and only sensible medical concepts". It is not a repository of every kind of information used in the practice of medicine. It aims to represent the underlying conceptual model of medicine that is shared across national boundaries, and hence can be usefully represented in a language- and application-independent way.

- **NCI Cancer Ontology [11, 12]**: The National Cancer Institute ontology provides definitions, synonyms, and other information on nearly 10,000 kinds of cancer and related diseases, 8,000 single agents and combination therapies, and a wide range of other topics related to cancer and biomedical research. Topics described in the ontology include diseases, drugs, chemicals, diagnoses, genes, treatments, anatomy, organisms and proteins.

- **Foundational Model of Anatomy Ontology (FMA) [13, 14]**: The FMA is a reference ontology for the domain of anatomy. It consists of 4 parts. The anatomy taxonomy classifies anatomical

entities according to the characteristics they share and by which they can be distinguished from one another. The anatomical structure abstraction specifies the part-whole and spatial relationships that exist between those entities. The anatomical transformation abstraction specifies the morphological transformation of those entities during prenatal development and the postnatal life cycle. Finally, the meta-knowledge specifies the principles, rules and definitions according to which classes and relationships in the other three components are represented. The FMA contains approximately 75,000 classes and over 120,000 terms. Over 2.1 million relationship instances from over 168 relationship types link the FMA's classes into a coherent symbolic model.

- **Gene Ontology (GO) [15, 16]**: The Gene Ontology is a major bioinformatics initiative to unify the representation of gene and gene product attributes across all species. The ontology covers three domains. The cellular component contains knowledge about the parts of a cell or its extracellular environment. The molecular function covers the elemental activities of a gene product at the molecular level, such as binding. Finally, the biological process models operations or sets of molecular events, with a defined beginning and end, which are pertinent to the functioning of integrated living units such as cells, tissues, organs and organisms. The GO is part of a larger classification effort namely the open biomedical ontologies (OBO) foundry. This is a joint effort to create ontologies for shared use across different biological and medical domains.

- **Ontology for Biomedical Investigations (OBI) [17, 18]**: OBI is an open access, integrated ontology for the description of biological and clinical investigations. OBI provides a model for the design of an investigation, the protocols and instrumentation used, the materials used, the data generated and the type of analysis performed on it. OBI is also a part of OBO.

List of abbreviations used

FMA: Foundational Model of Anatomy; Galen: Generalized Architecture for Languages, Encyclopaedias and Nomenclatures; GO: Gene Ontology; HL7: Health Level 7; IFOMIS: Institute for Formal Ontology and Medical Information Science; L&C: Language and Computing; NCI: National Cancer Institute; OBI: Ontology for Biomedical Investigations; OBO: Open Biomedical Ontologies; OWL: Ontology Web Language; SNOMED CT: Systematized Nomenclature of Medicine-Clinical Terms.

Acknowledgements

Femke Ongenae would like to thank the Institute for the Promotion of Innovation by Science and Technology in Flanders (IWT) for her Phd grant.

References

1. Matuszek C, Cabral J, Witbrock M, DeOliveira J: **An introduction to the syntax and content of Cyc**. In *Proceedings of the AAAI Spring Symposium on Formalizing and Compiling Background and Its Applications to Knowledge Representation and Question Answering: 27-29 March 2006; California, USA* 2006:44–49.
2. **OpenCyc** [http://www.opencyc.org].
3. Fellbaum C: *WordNet: An Electronic Lexical Database*. Cambridge, Massachusetts, USA: MIT Press 1998.
4. Van Gurp M, Decoene M, Holvoet M, dos Santos MC: **LinkBase®, a philosophically-inspired Ontology for NLP/NLU Applications**. In *Second International Workshop on Formal Biomedical Knowledge Representation (KR-MED): 8 November 2006; Baltimore, Maryland, USA*, National Center for Ontology Research (NCOR) and the Working Group on Formal (Bio-)Medical Knowledge Representation of the American Medical Informatics Association (AMIA) 2006:67–75.
5. Flett A, Dos Santos M, Ceuster W: **Some Ontology Engineering Processes and their Supporting Technologies**. In *Proceedings of the 13th International Conference on Knowledge Engineering and Management (EKAW): 1-4 October 2002; Siguenca, Spain* 2002:154–165.
6. **SNOMED CT** [http://www.ihtsdo.org/snomed-ct/].
7. Wasserman H, Wang J: **An Applied Evaluation of SNOMED CT as a Clinical Vocabulary for the Computerized Diagnosis and Problem List**. In *Proceedings of the annual American Medical Informatics Association (AMIA) Symposium: 8-12 November 2003; Washington, DC, USA* 2003:699–703.
8. Elkin PL, Brown SH, Husser CS, Bauer BA, Wahner-Roedler D, Rosenbloom ST, Speroff T: **Evaluation of the Content Coverage of SNOMED CT: Ability of SNOMED Clinical Terms to Represent Clinical Problem Lists**. *Mayo Clinic Proceedings* 2006, **81**(6):741–748.
9. **Galen Common Reference Model** [http://www.opengalen.org/].
10. Rector AL, Rogers JE, Zanstra PE, van der Haring E: **OpenGALEN: Open Source Medical Terminology and Tools**. In *Proceedings of the annual American Medical Informatics Association (AMIA) Symposium: 8-12 November 2003; Washington, DC, USA* 2003:982.
11. **NCI Cancer Ontology** [http://www.mindswap.org/2003/CancerOntology/].
12. Golbeck J, Fragoso G, Hartel F, Hendler J, Parsia B: **The National Cancer Institute's Thesaurus and Ontology**. *Journal of Web Semantics 2003* 2003, **1**:75–80.
13. **Foundational Model of Anatomy Ontology (FMA)** [http://sig.biostr.washington.edu/projects/fm/index.html].
14. Rosse C, Mejino JVL: **A reference ontology for biomedical informatics: the Foundational Model of Anatomy**. *Journal of Biomedical Informatics* 2003, **36**(6):478–500.
15. **Gene Ontology** [http://www.geneontology.org/].
16. Blake JA, Harris MA: **The Gene Ontology (GO) project: structured vocabularies for molecular biology and their application to genome and expression analysis**. *Current Protocols in Bioinformatics* 2008, **23**:7.2.1–7.2.9.
17. **Ontology for Biomedical Investigations** [http://obi-ontology.org/page/Main_Page].
18. Courtot M, Bug W, Gibson F, Lister AL, Malone J, Schober D, Brinkman R, RuttenBerg A: **The OWL of Biomedical Investigations**. In *Proceedings of the 4th International Workshop on OWL: Experiences and Directions (OWLED): 26-27 October 2008; Karlsruhe, Germany* 2008.
