# Supplementary material for: Towards computerizing intensive care sedation guidelines: design of a rule-based architecture for automated execution of clinical guidelines
Source: BMC Med Inform Decis Mak. 2010 Jan 18;10:3. doi: 10.1186/1472-6947-10-3 (PMC2823596; doi:10.1186/1472-6947-10-3)
Supplement: Additional file 3 — The 5 flow charts (UML diagrams) of the sedation guideline The zip (sedationGuidelines.zip) contains the 5 flow charts (UML diagrams) of the sedation guideline in pdf format. [file 1472-6947-10-3-S3.ZIP › ShortSedationReduction.pdf]

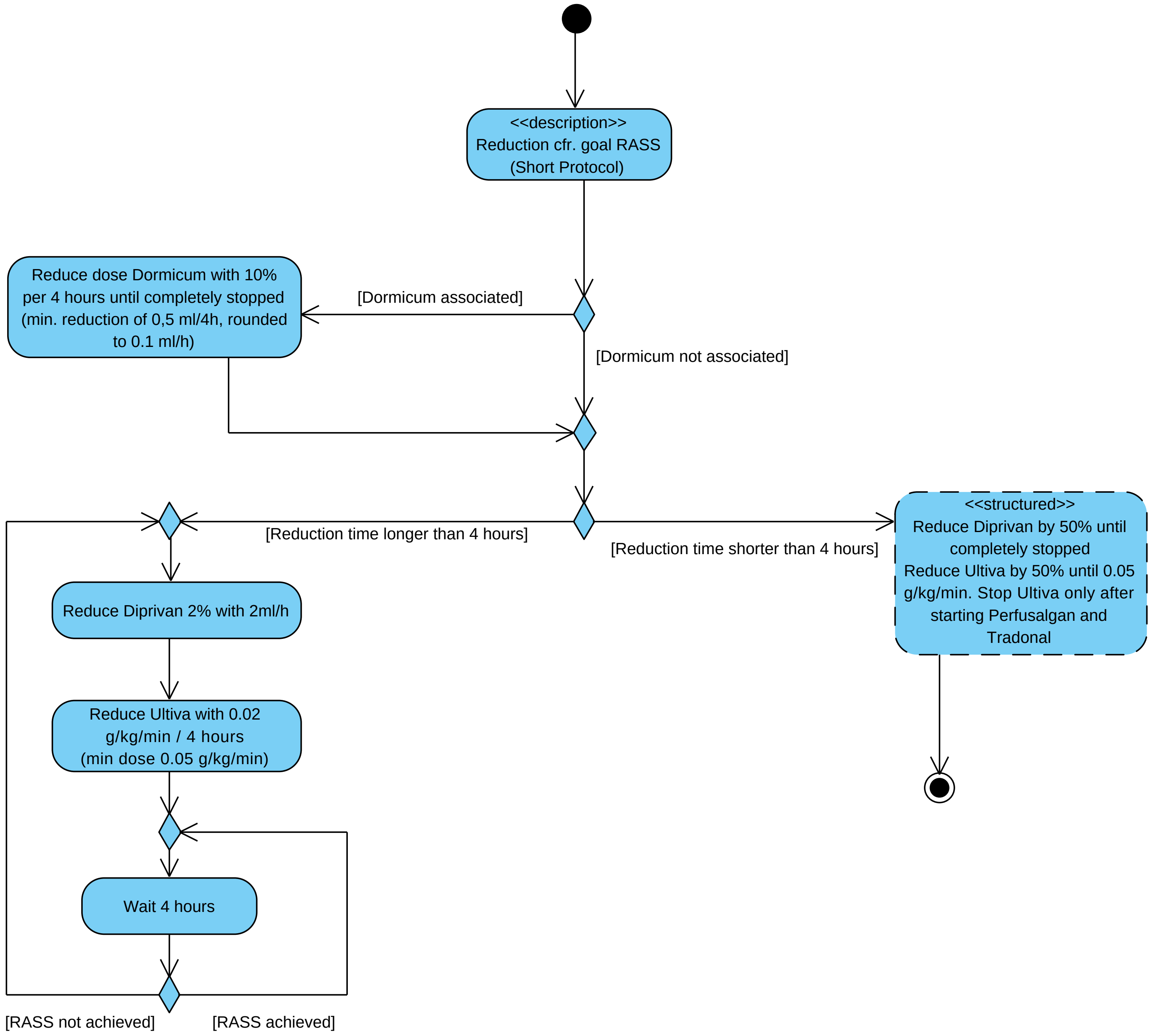
<<description>>
Reduction cfr. goal RASS
(Short Protocol)
[Dormicum associated]
Reduce dose Dormicum with 10% per 4 hours until completely stopped (min. reduction of 0,5 ml/4h, rounded to 0.1 ml/h)
[Dormicum not associated]
[Reduction time longer than 4 hours]
[Reduction time shorter than 4 hours]
<<structured>>
Reduce Diprivan by 50% until completely stopped
Reduce Ultiva by 50% until 0.05 g/kg/min. Stop Ultiva only after starting Perfusalgan and Tradonal
Reduce Diprivan 2% with 2ml/h
Reduce Ultiva with 0.02 g/kg/min / 4 hours
(min dose 0.05 g/kg/min)
Wait 4 hours
[RASS not achieved]
[RASS achieved]
