# Supplementary figures and images for: Towards computerizing intensive care sedation guidelines: design of a rule-based architecture for automated execution of clinical guidelines
Source: BMC Med Inform Decis Mak. 2010 Jan 18;10:3. doi: 10.1186/1472-6947-10-3 (PMC2823596; doi:10.1186/1472-6947-10-3)

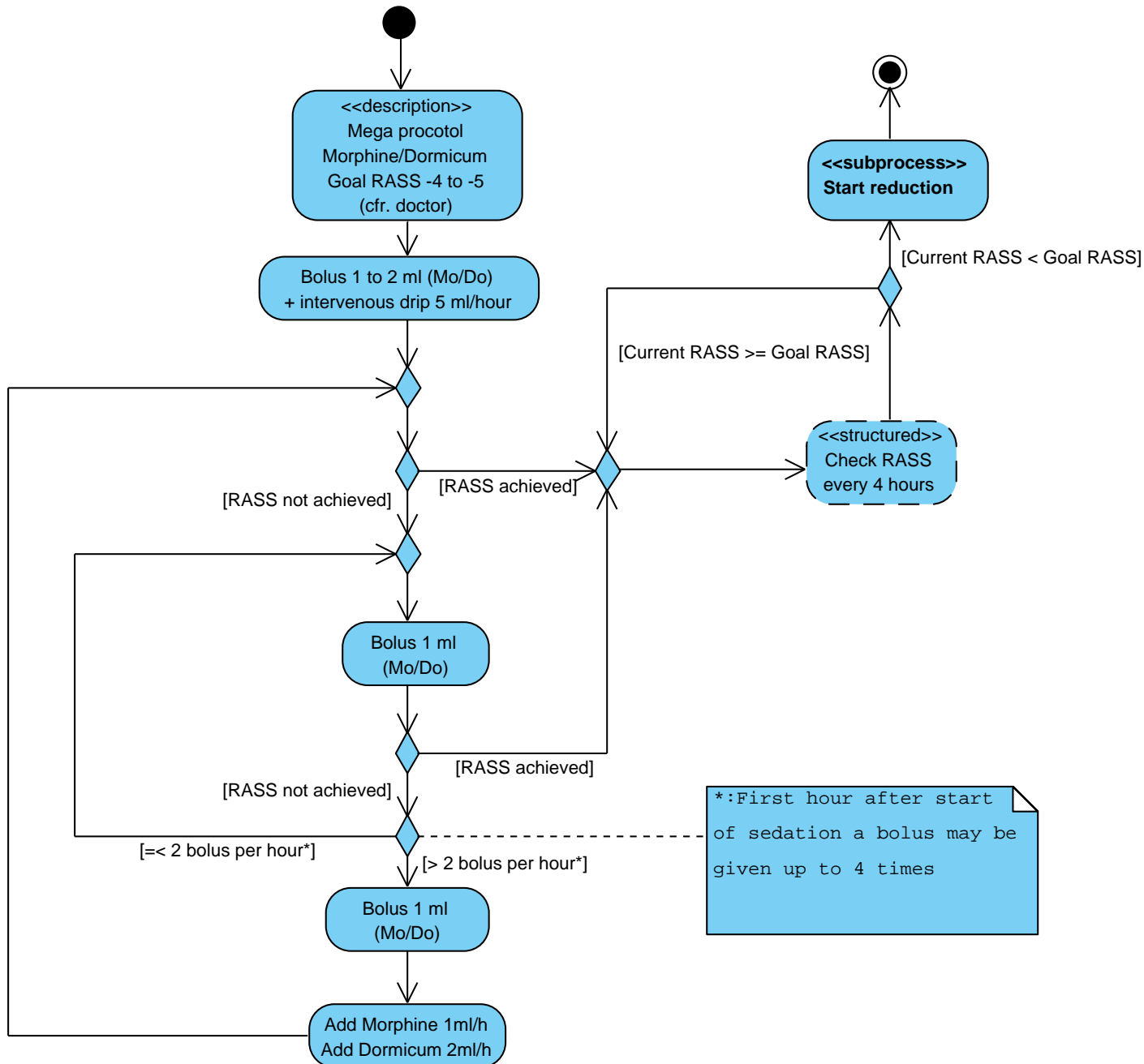

Supplement: Additional file 3 — The 5 flow charts (UML diagrams) of the sedation guideline The zip (sedationGuidelines.zip) contains the 5 flow charts (UML diagrams) of the sedation guideline in pdf format. [file 1472-6947-10-3-S3.ZIP › MegaSedation.pdf]

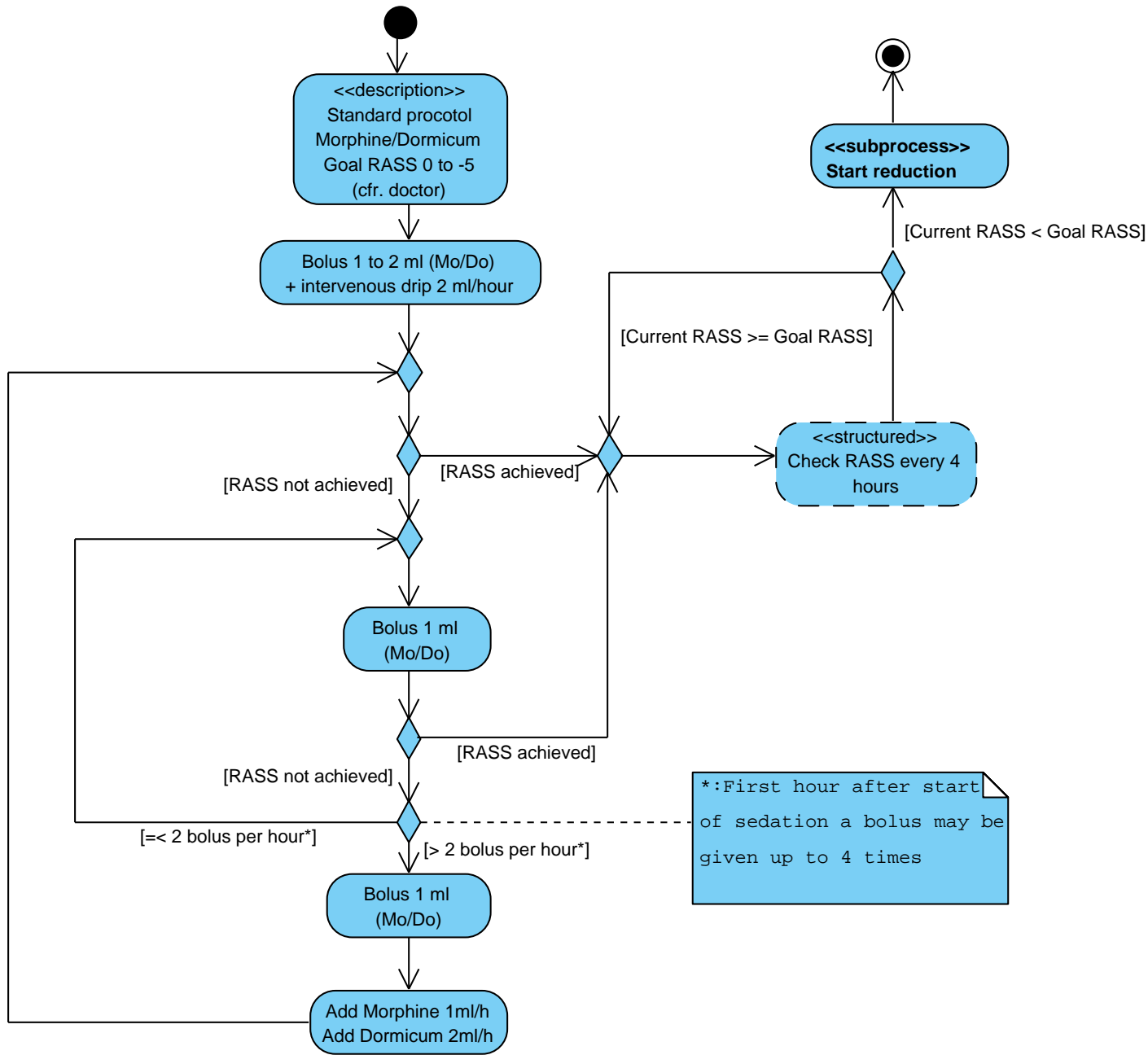

Supplement: Additional file 3 — The 5 flow charts (UML diagrams) of the sedation guideline The zip (sedationGuidelines.zip) contains the 5 flow charts (UML diagrams) of the sedation guideline in pdf format. [file 1472-6947-10-3-S3.ZIP › Sedation.pdf]

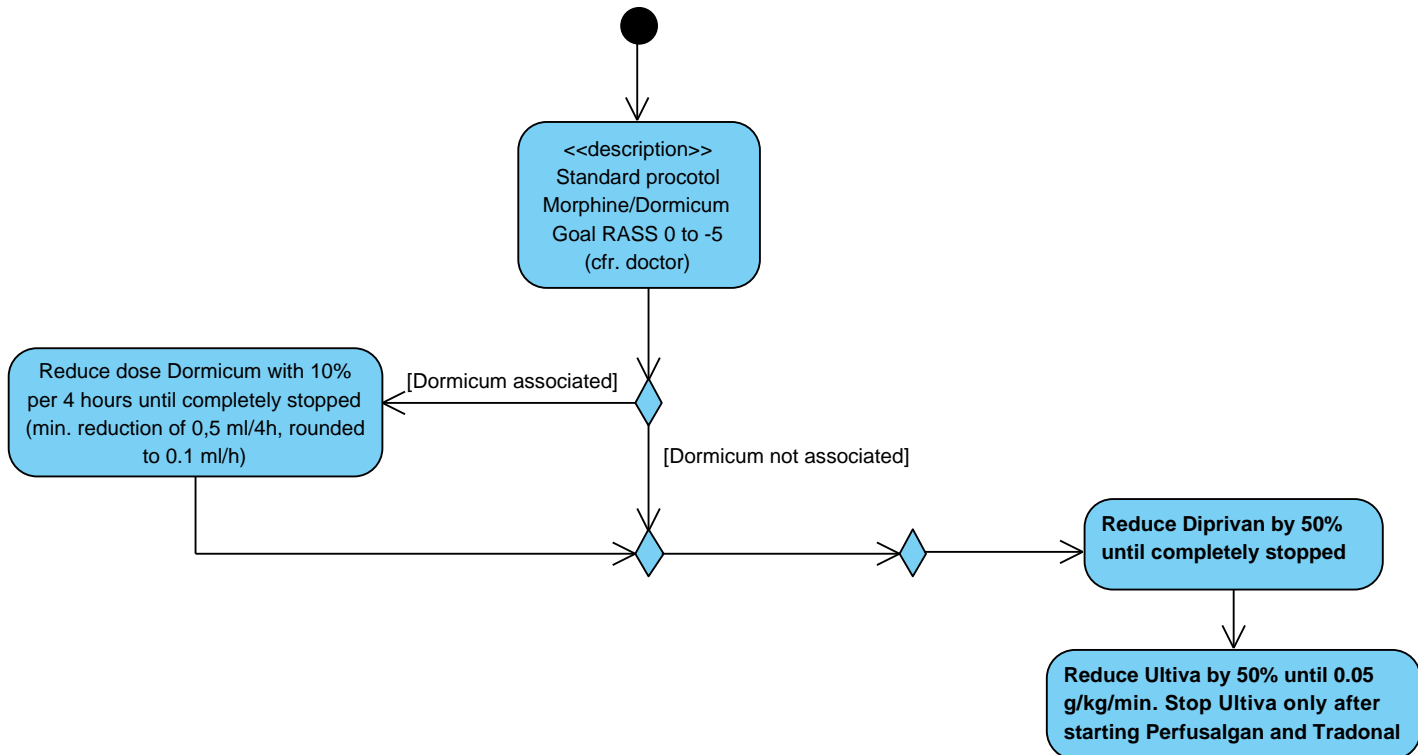

Supplement: Additional file 3 — The 5 flow charts (UML diagrams) of the sedation guideline The zip (sedationGuidelines.zip) contains the 5 flow charts (UML diagrams) of the sedation guideline in pdf format. [file 1472-6947-10-3-S3.ZIP › SedationReduction.pdf]

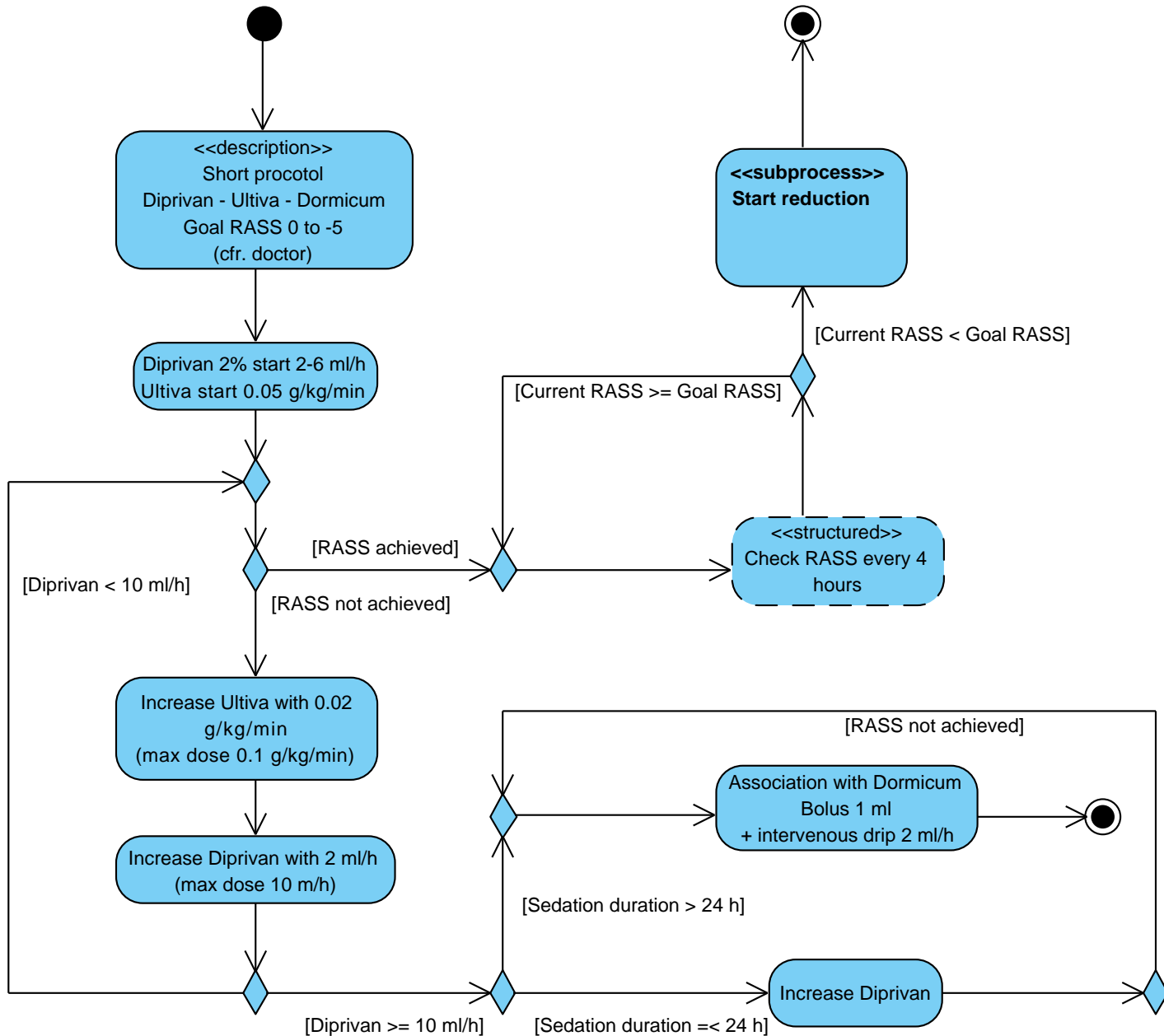

Supplement: Additional file 3 — The 5 flow charts (UML diagrams) of the sedation guideline The zip (sedationGuidelines.zip) contains the 5 flow charts (UML diagrams) of the sedation guideline in pdf format. [file 1472-6947-10-3-S3.ZIP › ShortSedation.pdf]
